# Insulin-like Growth Factor 1 Signaling in Mammalian Hearing

**DOI:** 10.3390/genes12101553

**Published:** 2021-09-29

**Authors:** Ángela García-Mato, Blanca Cervantes, Silvia Murillo-Cuesta, Lourdes Rodríguez-de la Rosa, Isabel Varela-Nieto

**Affiliations:** 1 Institute for Biomedical Research “Alberto Sols” (IIBm), Spanish National Research Council-Autonomous University of Madrid (CSIC-UAM), 28029 Madrid, Spain; agarciamato@iib.uam.es (Á.G.-M.); bcervantes@iib.uam.es (B.C.); smurillo@iib.uam.es (S.M.-C.); 2 Rare Diseases Networking Biomedical Research Centre (CIBERER), CIBER, Carlos III Institute of Health, 28029 Madrid, Spain; 3 La Paz Hospital Institute for Health Research (IdiPAZ), 28046 Madrid, Spain

**Keywords:** Ageing, AKT, GH, *IGF1* mutations, IGF system, inner ear, hearing loss, neurodegeneration, RAF, rare diseases

## Abstract

Insulin-like growth factor 1 (IGF-1) is a peptide hormone belonging to the insulin family of proteins. Almost all of the biological effects of IGF-1 are mediated through binding to its high-affinity tyrosine kinase receptor (IGF1R), a transmembrane receptor belonging to the insulin receptor family. Factors, receptors and IGF-binding proteins form the IGF system, which has multiple roles in mammalian development, adult tissue homeostasis, and aging. Consequently, mutations in genes of the IGF system, including downstream intracellular targets, underlie multiple common pathologies and are associated with multiple rare human diseases. Here we review the contribution of the IGF system to our understanding of the molecular and genetic basis of human hearing loss by describing, (i) the expression patterns of the IGF system in the mammalian inner ear; (ii) downstream signaling of IGF-1 in the hearing organ; (iii) mouse mutations in the IGF system, including upstream regulators and downstream targets of IGF-1 that inform cochlear pathophysiology; and (iv) human mutations in these genes causing hearing loss.

## 1. The Insulin-Like Growth Factor System (IGF System)

**Figure 1:**
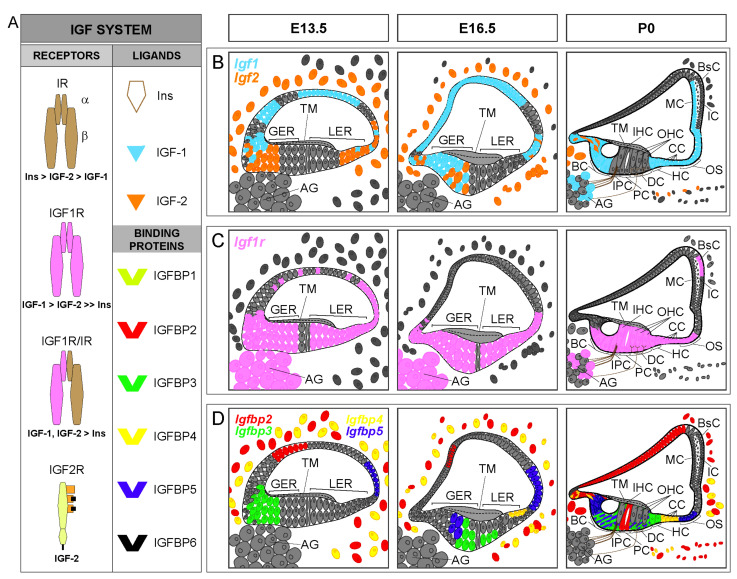
IGF system expression patterns in the developing cochlea. (**A**). Schematic representation of the IGF system and receptors binding affinity. The IGF system consists of three ligands (insulin, IGF-1 and IGF-2), transmembrane receptors [insulin receptor (IR), IGF receptors type 1 (IGF1R) and 2 (IGF2R) and hybrid receptor (IGF1R/IR)], and six high affinity IGF-binding proteins (IGFBPs 1–6). (**B**–**D**) Cartoons of the mouse organ of Corti at embryonic day E13.5, E16.5 and postnatal day P0, showing available cochlear IGF system expression patterns: *Igf1* (blue), *Igf2* (orange), *Igf1r* (pink), *Igfbp2* (red), *Igfbp3* (green), *Igfbp4* (yellow) and *Igfbp5* (purple). In (**D**), the expression of *Igfbp3* in supporting cells overlaps with the expression of *Igfbp5* at P0 (green cells with purple lines). GER, greater epithelial ridge; LER, lesser epithelial ridge; IHC, inner hair cells; OHC, outer hair cells; BC, border cells; IPC, inner phalangeal cells; PC, pillar cells; DC, Deiters’ cells; HC, Hensen’s cells; CC, Claudius’ cells; OS, outer sulcus cells; AG, auditory ganglion; TM, tectorial membrane; MC, marginal cells; IC, intermediate cells; BsC, basal cells. Data have been compiled from: (**A**) [1,2,3] and (**B**–**D**) [8,9,10,11,12,13,16].

Insulin-like growth factors (IGFs) are evolutionarily conserved peptides of the insulin family with key roles in developmental growth, cellular differentiation and homeostasis [1]. The mammalian IGF system is a complex network of signaling pathways mainly comprising: (i) three ligands–insulin, IGF-1, and IGF-2; (ii) three transmembrane receptors – insulin receptor (IR) and IGF receptors type 1 (IGF1R) and type 2 (IGF2R); and (iii) six soluble, high-affinity IGF-binding proteins (IGFBPs 1–6), which bind IGFs but not insulin [1,2,3] (Figure 1A).

Insulin is specifically produced by the β-cells of the islets of Langerhans in the pancreas and is secreted into blood circulation in response to elevated glucose levels [4]. By contrast, IGF-1 and IGF-2 are produced by multiple cell types, where they act in a paracrine/autocrine manner [1]. The biosynthesis of IGF-1 and IGF-2 occurs mainly in the liver, where hepatic IGF and the acid-labile subunit (ALS) protein are synthesized, and enter circulation as ternary complexes of 140–150 kDa in association with IGFBP3 or IGFBP5. Circulating IGF-1 and IGF-2 are generally found in these ternary complexes, which prolong their circulating half-life, although they can exist in binary complexes with all six IGFBPs or as free forms, which are available to interact with cell surface receptors [1,2,3].

IGF-1 secretion by the liver is stimulated by growth hormone (GH), also known as somatotropin, which is produced and secreted in pulsatile bursts by the somatotroph cells of the pituitary gland and has a short circulating half-life. *GH* gene expression is controlled, among others, by the pituitary-specific transcription factors POU class 1 homeobox 1 (POU1F1) and PROP paired-like homeobox 1 (PROP1), which are also implicated in adenohypophysis development and associated with hypopituitarism [5,6]. Two main hypothalamic hormones are known to modulate GH secretion: GH-releasing hormone (GHRH), which stimulates the production and secretion of GH by binding to the GHRH receptor (GHRHR), and somatostatin release-inhibiting factor (SRIF or somatostatin), which inhibits the release of GH. Binding of GH to its cognate receptor (GHR) induces receptor dimerization, triggering the activation of intracellular signal transduction through receptor-associated Janus kinase 2 (JAK2), which in turn activates (by phosphorylation) Signal transducer and activator of transcription 5 (STAT5), a mechanism crucial in controlling liver *IGF-1* gene expression [7].

Both *Igf1* and *Igf2* (encoding IGF-1 and IGF-2, respectively) are expressed in the mammalian cochlea during embryonic development with a specific regional pattern (Figure 1B). *Igf1* expression in the developing mouse cochlea (from E13.5 to P0) is localized mainly in the medial edge of the greater epithelial ridge, which includes the unique row of inner hair cells, and in the domain corresponding to the future stria vascularis; expression is weaker in the areas that will form Reissner’s membrane, the spiral limbus and the outer sulcus. *Igf2* expression overlaps partially with *Igf1* expression, as it is localized in the medial region of the cochlear duct, and is also found in the mesenchyme that surrounds the cochlear duct, but it is not expressed in the stria vascularis [8,9]. In the first weeks after birth (from P1 to P20), *Igf1* expression in the early postnatal cochlea is mainly restricted to the marginal cells of the stria vascularis, the spiral limbus, the medial greater epithelial ridge cells, the supporting cells of the organ of Corti (inner sulcus, Claudius’ and Hensen’s cells), and a subpopulation of neurons of the spiral ganglion [8,10,11,12]. Similar to *Igf1*, *Igf2* expression remains high during development and falls dramatically after birth (from P1 to P30), becoming localized in the interdental cells and in the medial greater epithelial ridge cells [8,12,13,14]. In the adult cochlea, *Igf1* expression is reduced relative to embryonic stages [14] and it is mainly localized in Deiters’ cells, whereas *Igf2* expression is predominant in the pillar cells [8,15]. The expression of *Igf1* and *Igf2* can still be detected in 12-month-old mouse cochleae [14]. In contrast to the detailed information reported on IGFs expression in the developing and early postnatal cochlea, the information on the postnatal expression of the factors is scarce. Similarly, there is no sufficient information available on the cochlear expression of insulin genes as to define expression patterns.

IGF1R and IR are receptor tyrosine kinases. IR has two alternatively spliced isoforms in mammals (IR-A and IR-B). While the physiological roles are not fully clarified, their different binding affinities for IGFs appear to be determinant [17]. IGF1R and IR monomers can form homo- or heterodimers at the cell membrane. Each monomer is formed by two disulfide-linked polypeptide chains: the extracellular α-chain containing the ligand-binding domain, and the β-chain containing extracellular, transmembrane and intracellular domains, with the latter harboring the intrinsic tyrosine kinase activity. IGF1R and IR (both A and B isoforms) share 70% homology in their amino acid sequences and over 80% homology in the intracellular kinase domain [17,18], but they mediate distinct physiological functions. IGF1R and hybrid IGF1R/IR can bind with high affinity to IGF-1 and IGF-2, and with lower affinity to insulin. As expected, IR-A and IR-B have greater affinity for insulin than for IGF-1, although IGF-2 binds to IR-A with an affinity similar to that of insulin [1,17,19]. In contrast to IGFR1, IGF2R (also known as the mannose-6-phosphate receptor) lacks the tyrosine kinase activity of IGF1R and IR. IGF2R mainly binds IGF-2 at the cell surface where it is cleared by receptor-mediated endocytosis and lysosomal degradation, thus controlling IGF-2 extracellular availability [20]. IGF2R has been reported to have a role in signal transduction through its interaction with G-proteins [20,21].

*Igf1r* is also highly abundant in the developing and postnatal mammalian cochlea (Figure 1C). It is expressed ubiquitously within the cochlear duct along embryonic development, but its expression is stronger in the pro-sensory domain, the greater epithelial ridge, the presumptive Reissner’s membrane and the spiral ganglion. Following birth, *Igf1r* expression is restricted to the inner and outer spiral sulcus, the medial greater epithelial ridge cells, the supporting cells, the outer and inner hair cells and the basal cells of the stria vascularis, showing a complementary pattern to that of *Igf1* [8,9,11,12]. *Igf1r* gene expression diminishes significantly from embryonic day E15.5 to postnatal day P5, but then increases slightly from P15 onwards [8,13,14], and is mainly localized in the outer hair cells [15]. *Igf1r* has been detected in the mouse cochlea at 12 months of age, with levels similar to those found at one month of age [14].

IGFBPs are essential for the transport and action of IGFs. IGFBPs control IGF-1 and IGF-2 bioavailability in the blood circulation and at the cell surface by prolonging their half-life, regulating their clearance, and providing a tissue-specific localization [1,2,3]. IGFBPs form ternary complexes with ALS and IGF-1 that extend the half-life of the circulating factor [1]. IGFBPs most studied are the high affinity 1–6, although there are up to 11 structurally, related proteins with low affinity for IGF-1, whose role is under study [3,22].

IGFBPs also inhibit IGF actions by preventing its binding to IGF1R. IGFBPs activity is controlled by tissue-specific proteases that indirectly increase the bioavailability of IGFs and potentiate their actions, as proteolyzed IGFBPs bind with low or no affinity to IGFs. IGFBPs also have IGF-independent roles in cell proliferation, differentiation, survival and migration, which involves binding to cell surface receptors, activation of intracellular signaling pathways, and nuclear translocation to control gene expression [2,3].

The expression of *Igfbp2–5* has been principally studied during the embryonic development of the cochlea (Figure 1D) and scarcely in adult mice [8,16]. During mouse cochlear development, *Igfbp2* is expressed in an area that will form Reissner’s membrane, and in the periotic mesenchymal cells surrounding the cochlear duct. *Igfbp2* is also localized in the pillar cells at P0 [16], and its expression is mainly observed in the inner phalangeal, interdental, inner and outer pillar and in Deiters’ cells at P1 [12]. *Igfbp3* and *Igfbp5* have a corresponding expression pattern in the pro-sensory domain in the developing cochlea. At P0, both *Igfbp3* and *Igfbp5* are expressed in supporting cells, and *Igfbp5* is also found in Kölliker’s organ and in Claudius’ cells [16]. At P1, *Igfbp3* and *Igfbp5* are broadly expressed in the supporting cells (Deiters’, inner and outer pillar and inner phalangeal cells), and in the lateral greater epithelial ridge cells [12]. The expression pattern of *Igfbp4* during cochlear development is similar to that of *Igfbp2*, although it is also expressed in the presumptive Claudius’ cells. The expression of *Igfbp4* is maintained in Claudius’ cells at perinatal ages, and it is also found in the cochlear modiolus, the spiral limbus and the lateral cochlear wall [12,16]. In the adult mouse cochlea, the expression of *Igfbp2–4* is mainly restricted to the pillar cells, whereas *Igfbp5* is found in hair cells, Deiters’ cells and pillar cells. The study of genes for IGFBP expression along aging has shown that it is dramatically reduced in comparison with embryonic stages [8,14,15], although it is maintained in 12-month-old mice [14]. The cochlear expression of genes for other high- or low- affinity IGFBPs has not been reported, although single-cell RNAseq is providing new data that remain are still to be fully understood (gEAR website; https://umgear.org/, accessed on 14 May 2021).

## 2. IGF-1 Signaling Network

**Figure 2:**
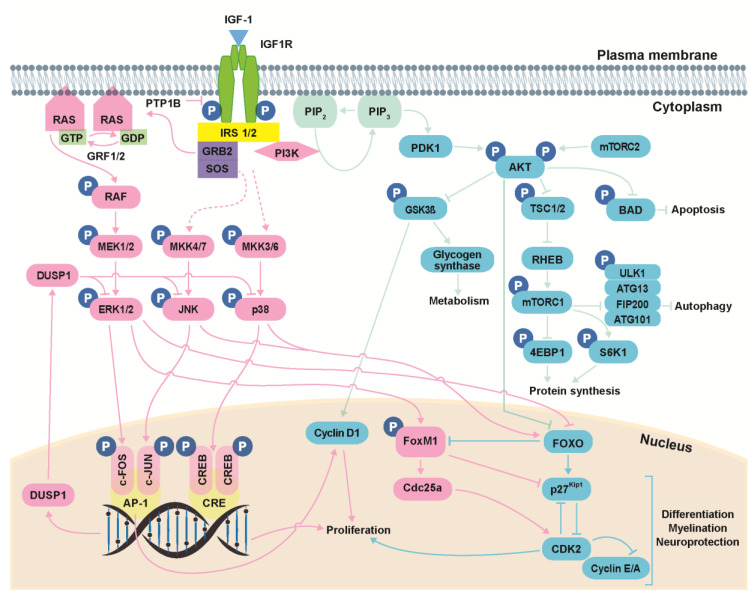
IGF-1 signaling. Activation of IGF1R can result in signaling via three main pathways: PI3K/AKT, ERK/MAPK and stress kinases (p38 and JNK). The PI3K/AKT pathway modulates cell survival, autophagy, protein synthesis and glucose metabolism; the ERK/MAPK pathway regulates cell growth and differentiation, among other processes; and the stress kinase (p38 and JNK) cascades regulate stress response, proliferation, differentiation, migration and survival. Kinase actions are strictly regulated by phosphatases. PTP1B, protein tyrosine phosphatase 1B; IRS, insulin receptor substrate; GRB2, growth factor receptor-bound protein 2; SOS, son of sevenless; RAS, rat sarcoma; RAF, rapidly accelerated fibrosarcoma; ERK, extracellular signal-regulated kinase; MAPK, mitogen-activated protein kinase; JNK, c-JUN N-terminal kinases; PDK1, phosphoinositide-dependent protein kinase 1; PIP2, phosphatidylinositol 4,5-bisphosphate; PIP3, phosphatidylinositol (3,4,5)-trisphosphate; PI3K, phosphatidylinositol 3-kinase; AKT, thymoma viral proto-oncogene; mTORC1, mammalian target of rapamycin complex 1; mTORC2, mammalian target of rapamycin complex 2; GSK3β, glycogen synthase kinase-3β; TSC1, tuberous sclerosis complex-1; TSC2, tuberous sclerosis complex-2; RHEB, RAS homolog enriched in brain; ULK1, unc-51-like kinase 1; ATG13, mammalian autophagy-related gene 13; FIP200, focal adhesion kinase family-interacting protein of 200 kDa; ATG101, mammalian autophagy-related gene 101; S6K1, p70S6 Kinase 1; 4EBP, eukaryotic translation initiation factor 4E-binding protein; FOXO, forkhead box O; FOXM1, forkhead box protein M1. Data have been compiled from [18,19,23,27,29,30,35,36,37,38].

The biological actions of IGF-1 are primarily mediated by binding to its high-affinity receptor, IGF1R (Figure 2). Binding of IGF-1 to the α-subunits of IGF1R triggers receptor activation through trans-autophosphorylation of tyrosine residues in the β-subunits [19]. Activated IGF1R then recruits and activates (by phosphorylation) several docking proteins, including the insulin receptor substrates (IRS 1–4) and the SRC homology collagen (SHC) protein, which possesses a phosphotyrosine-binding domain that recognizes the –NPXY– motif of IGF1R. The phosphorylated tyrosine residues of these docking proteins are recognized by signaling molecules such as GRB2 and PI3K, which contain a SRC homology 2 (SH2) domain, leading to the activation of a network of signaling pathways [18,19]. Protein tyrosine phosphatase 1B (PTP1B) is a negative modulator of IGF1R activation that directly interacts with IGF1R and dephosphorylates the tyrosine residues within its catalytic domain, leading to receptor inactivation [23]. Analysis of IGF1R docking proteins and their regulation in the mouse cochlea has revealed that *Irs1* and *Irs2* activation is essential for cochlear development and hearing function [24,25]. *Ptpn1,* which encodes for PTP1B, is also expressed in the mouse cochlea, where it acts as a key modulator of IGF1R-mediated signaling [24].

One of the key pathways activated downstream of IGF1R is the extracellular signal-regulated kinase (ERK) mitogen-activated protein kinase (MAPK) cascade, whose activation is regulated by growth factor receptor-bound protein 2 (GRB2) [26]. Among other processes, this pathway modulates early gene expression to control cell growth and differentiation [27]. Other well-defined MAPK pathways controlled by IGF-1 include the p38 stress kinases and the c-JUN N-terminal kinases (JNK) [28], which are activated in response to environmental stress (e.g., UV irradiation, oxidative stress, hypoxia, inflammatory cytokines or nutrient deprivation) to coordinately regulate proliferation, differentiation, migration and survival [29]. Finally, the third main pathway activated downstream of IGF1R is controlled by the 85-kDa regulatory subunit of phosphatidylinositol 3-kinase (PI3K), which promotes the activation of PI3K–AKT (thymoma viral proto-oncogene) signaling to modulate cell survival, autophagy, protein synthesis and glucose metabolism [30].

As an alternative form of signaling, activated IGF1R can also translocate to the nucleus after SUMOylation of the lysine residues of its β-subunits [31], likely occurring by clathrin-mediated endocytosis [32]. IGF1R nuclear translocation has been primarily described in cancer cells [31,32], but has also been detected in cell lines and primary human fibroblast cultures [33]. Once in the nucleus, IGF1R binds to putative enhancer regions to modulate the transcription of specific genes, leading to elevated levels of cyclin D1, cyclin A and CDK2, as well as to the downregulation of p27^Kip1^ [34].

### 2.1. RAF/MEK/ERK Cascade

By engaging IGF1R, IGF-1 modulates the activity of the RAF/MEK/ERK MAPK cascade through the interaction between the phosphotyrosine residues of the docking proteins (IRS1, IRS2 or SHC) and the SH2 domain of GRB2 [19]. The latter interaction mediates the membrane translocation of Son of sevenless (SOS), a guanine nucleotide-exchange factor for the small GTPase RAS (Rat sarcoma). This interaction triggers RAS activation, which in turn activates the rapidly accelerated fibrosarcoma (RAF) proteins (A-RAF, B-RAF and C-RAF). RAFs are serine kinases that form homo- and heterodimers in their kinase domain, and activate (by phosphorylation) the dual-specificity kinases MEK1 (MAPK ERK kinase 1) and MEK2. These proteins, in turn, activate ERK1 and ERK2 through phosphorylation on threonine and tyrosine residues. ERK1/2 are the main effectors of the cellular processes controlled by the RAF/MEK/ERK pathway, such as cell proliferation. In this context, ERK1/2 promote the G1/S-phase transition of the cell cycle. ERK1/2 also have a role in cell survival by inhibiting the intrinsic apoptosis pathway and stimulating the expression of antiapoptotic proteins. Beyond these functions, ERK1/2 are also involved in the regulation of growth, differentiation, metabolism and migration [27,39].

All three RAF kinases are expressed and active in the developing and postnatal mouse cochlea and show similar gene expression levels at both developmental stages, although their protein levels drop dramatically after birth [40]. Single-cell expression analysis in the mouse cochlea revealed that all three *Raf* genes are broadly distributed within the organ of Corti, with *CRaf* being the most abundant. The expression of *Mapk1* (ERK2) and *Mapk3* (ERK1) follows a similar pattern to that of the *Raf* genes in the postnatal mouse cochlea [12].

During mouse cochlear development, ERK is preferentially activated in the lateral-roof side of the cochlear duct, including the regions of the outer sulcus and stria vascularis. At E12.5, ERK activation is higher in the apex tip of the roof side and propagates from the apex to the base of the floor side [41], suggesting that ERK activation is necessary to drive the spiral morphogenesis of the cochlear duct. 

ERK activation is observed in the mouse cochlea in response to acoustic trauma [42,43,44] and to aminoglycoside treatment [45]. Acutely after noise exposure, activated ERK is found in the spiral ligament, in the neurosensory epithelium of the organ of Corti (inner and outer hair cells, inner pillar cells and Deiters’ cells), in the lateral wall, in the spiral ganglion neurons and in the modiolus, independently of *de novo* synthesis [42,43,44]. As ERK upregulation occurs immediately after noise trauma, it is likely to be a protective mechanism rather than a harmful response. However, neomycin-induced hair cell loss in a mouse ototoxicity model was associated with ERK phosphorylation [45], suggesting that neomycin promotes hair cell death through ERK pathway activation in this model. Accordingly, further work is needed to elucidate whether phosphorylated ERK contributes to or protects against hair cell death.

### 2.2. Stress Kinase (p38 and JNK) Cascades

Both p38 and JNK MAPKs are activated in response to cellular stress through dual phosphorylation on threonine and tyrosine residues in their activation loops (Figure 2). Among the MAPK kinases (MKKs) that catalyze this phosphorylation reaction, MKK3/6 are thought to be the main activators of p38 [36] and MKK4/7 are specific for JNK [37]. P38 is critical in immune and inflammatory responses by stimulating proinflammatory cytokine production, and it is also involved in cell proliferation by favoring cell cycle arrest at G1/S and G2/M phases through downregulating cyclins and upregulating CDK inhibitor expression. JNK also has a role in cell proliferation but, unlike p38, it typically promotes the transcription of genes that stimulate the cell cycle, such as cyclin D1. Both stress kinases are involved in the induction of apoptosis through p53 activation [29].

The study of the gene expression of p38 (*Mapk11–14*) and JNK (*Mapk8–9*) kinases in the mouse cochlea has revealed that both are expressed during embryonic development [8], postnatally [12] and along aging [46]. Analysis of single-cell expression of the postnatal organ of Corti revealed that *Mapk14* (p38α) and *Mapk12* (p38γ) are broadly expressed within the cochlear epithelium, *Mapk13* (p38δ) is the more abundant p38 isoform, and *Mapk11* (p38β) is barely expressed. Regarding JNK, *Mapk9* (JNK2) is the predominant isoform, and is expressed in supporting cells of the organ of Corti [12].

Both stress kinases have been shown to be involved in noise [42,44] and aging [47] responses in the adult cochlea. Levels of activated p38 and JNK were found to increase acutely after noise exposure, mainly in the spiral ligament, the sensory and supporting cells of the organ of Corti, the stria vascularis and the spiral neurons, and this was still evident in the spiral neurons 48 h post-exposure [42,44]. Activation of both stress kinases has also been observed in outer hair cells and spiral ganglion cells in a mouse model of aging [47].

As MAPKs are activated by phosphorylation, their actions are inhibited by the dual-specificity MAPK phosphatases (MKPs), which specifically dephosphorylate threonine and tyrosine residues of the phosphorylated MAPKs to control their actions. MKPs can be divided into three groups based on their gene structure, sequence homology, subcellular localization and substrate specificity: (i) inducible nuclear MPKs (MKP1 [DUSP1], PAC1 [DUSP2], MKP2 [DUSP4] and HVH3 [DUSP5]), which target all MAPKs with different substrate affinities based on the cell type and context; (ii) cytoplasmic, ERK-specific MKPs (MKP3 [DUSP6], MKPX [DUSP7] and MKP4 [DUSP9]); and (iii) nuclear and cytoplasmic MKPs (DUSP8, MKP5 [DUSP10] and MKP7 [DUSP16]), which specifically dephosphorylate the stress-activated p38 and JNK MAPKs [35].

Analysis of the gene expression of MKPs in the mouse cochlea has revealed that *Dusp6*, *Dusp7* and *Dusp9* are expressed during mouse inner ear development [48], demonstrating that ERK is crucial in embryonic stages. A comprehensive analysis of *Dusp* expression by Celaya and colleagues [46] demonstrated that *Dusp1, Dusp2, Dusp4, Dusp5*, *Dusp6–8*, *Dusp10* and *Dusp16* are expressed in the postnatal mouse cochlea. Moreover, the authors found that *Dusp1* expression was age-regulated, and increased with aging, and that DUSP1 levels increased acutely after noise exposure [46,49]. It has also been reported that *Dusp1*, *Dusp5* and *Dusp6* expression is upregulated in the mouse cochlea after noise trauma [50].

### 2.3. PI3K-AKT Pathway

The interaction between the SH2 domain of the 85-kDa regulatory subunit of PI3K and the phosphorylated tyrosine residues of IRS1 and IRS2 triggers PI3K recruitment to the cell membrane and activation of its 110-kDa catalytic subunit. Activated PI3K, in turn, phosphorylates PI4,5P_2_ to generate PI3,4,5P_3_, which is required to transfer inactive AKT and one of its activators, PDK1 (phosphoinositide-dependent protein kinase 1), to locations in the membrane where PI3,4,5P_3_ accumulates. AKT is a serine/threonine kinase that is fully activated by dual phosphorylation on threonine 308 residue by PDK1 and serine 473 by the mammalian target of rapamycin complex 2 (mTORC2) [19,30]. Activated AKT is a signaling node that regulates multiple downstream signaling pathways involved in cell survival and cellular homeostasis by regulating anabolism and autophagy [30].

*Akt1–3* are expressed in the mouse cochlea during embryonic development [8] and after birth [12,51], and all three *Akt* isoforms are broadly distributed within the cell types that comprise the organ of Corti [12]. Among them, *Akt2* is the predominant isoform, and is expressed primarily in the organ of Corti and the stria vascularis [51].

The PI3K-AKT pathway is known to be involved in the onset of age-related hearing loss (ARHL). The levels of PI3,4,5P_3_ in the inner and outer hair cells, Deiters’ cells and pillar cells in the mouse cochlea, have been reported to decrease with aging concomitant with a reduction of activated AKT in outer hair cells [52]. Similar results were reported in a model of kanamycin- [53] and gentamicin-induced damage [54].

Glycogen synthase kinase-3β (GSK3β), which inhibits glycogen synthase, is one of the physiological targets of AKT, which phosphorylates GSK3β on serine 9 to increase glycogen synthesis and storage [30]. In addition to regulating glycogen storage, GSK3β also targets cyclin D1 for proteasome-mediated degradation. Accordingly, GSK3β inhibition by AKT leads to an increase in cyclin D1 levels, favoring G1/S cell cycle transition [19].

GSK3β is expressed in the supporting cells of the organ of Corti in the developing mouse cochlea, and its expression declines after birth, although it is still detected in inner pillar, Hensen’s and Claudius’ cells [55]. Expression of the gene for GSK3β during cochlear development is determinant for cell fate determination and patterning of the cochlear duct [55].

The PI3K-AKT pathway also modulates the activity of mTORC1. MTOR is a serine/threonine kinase that regulates cell growth and metabolism in response to environmental signals, and promotes protein, lipid and nucleotide synthesis but inhibits autophagy. mTOR is also a signaling node and interacts with multiple proteins to form two complexes: mTORC1 and mTORC2 [38]. AKT phosphorylates and inhibits the heterodimer formed by tuberous sclerosis complex-1 (TSC1; also known as hamartin) and TSC2 (also known as tuberin). This complex is an upstream negative regulator of mTORC1 and maintains the binding of RAS homolog enriched in brain (RHEB), which is necessary for mTOR activation, to GDP and, hence, is inactivated. Inactivation of this complex by AKT abrogates its GTPase-activating protein activity, thus preserving RHEB bound to GTP and favoring mTORC1 activation [30]. Once activated, mTORC1 promotes protein synthesis mainly by phosphorylating the p70S6 kinase 1 (S6K1) and the eukaryotic translation initiation factor 4E (eIF4E)-binding protein (4EBP). The phosphorylation of S6K1 leads to an increase in mRNA biogenesis and stimulates protein translation. Phosphorylation of 4EBP prevents its binding to eIF4E, enabling the initiation of protein translation [38]. Beyond its role in promoting anabolism, mTORC1 inhibits catabolism by phosphorylating ULK1 (unc-51-like kinase 1), a kinase which forms the autophagy initiation complex along with ATG13 (mammalian autophagy-related gene 13), FIP200 (focal adhesion kinase family-interacting protein of 200 kDa) and ATG101 [38]. The phosphorylation of ULK1 by mTORC1 [56] destabilizes the ULK1/ATG13/FIP200/ATG101 complex and reduces ULK1 kinase activity, leading to the inhibition of the kinase complex required for autophagy initiation [38].

Leitmeyer and colleagues [57] demonstrated that both mTORC1 and mTORC2 are present in the postnatal rat cochlea. Moreover, they found that inhibition of mTOR by rapamycin promoted hair cell loss and reduced the number and length of the spiral ganglion neurites. These findings strongly suggest that mTOR plays a key role in the survival of hair cells and modulates neuritogenesis of spiral ganglion neurons and dendrite formation in the mammalian cochlea. Several autophagy-related genes are expressed during cochlear development and throughout life in mice, and their expression increases with age. Moreover, autophagic flux is upregulated from perinatal ages to adulthood, concomitant with the functional maturation of the organ of Corti. Hence, autophagy plays a key role in the development and maturation of the mouse cochlea, and the regulation of this process by IGF-1 appears to be an essential mechanism for correct hearing function [13,58].

Activation of the aforementioned three main signaling pathways by IGF-1 controls the expression of specific genes through the regulation of transcription factors, such as the forkhead box O (FOXO) family, which includes FOXO1, 3, 4 and 6. Activated ERK1/2 and AKT promote the translocation of FOXO factors from the nucleus to the cytoplasm, where they are degraded, leading to the inhibition of the expression of pro-apoptotic genes and genes involved in cell cycle arrest. By contrast, p38 and JNK stress kinases induce FOXO nuclear localization and the expression of their target genes. One of the key downstream targets of FOXO3 is FOXM1 (forkhead box protein M1), a transcription factor ubiquitously expressed in proliferating cells whose activity and expression is suppressed by FOXO3. FOXM1 promotes cell proliferation by modulating the transcription of genes involved in G1/S and G2/M progression. Both FOXO3 and FOXM1 modulate the activity of the cyclin-dependent kinase inhibitor p27^Kip1^, but with opposing effects: while FOXO3 promotes p27^Kip1^ transcription, leading to cell cycle arrest, FOXM1 blocks nuclear localization of p27^Kip1^ and enhances its degradation, promoting cell proliferation [30,59]. 

FOXO3 has been shown to be involved in the ototoxic side-effects of amikacin, an aminoglycoside antibiotic used for severe bacterial infections. Amikacin treatment promotes phosphorylation of FOXO3 in the organ of Corti, spiral ganglion and stria vascularis, leading to an increase in the expression of pro-apoptotic proteins and a reduction in the levels of anti-apoptotic mediators [60]. It has been reported that both transcripts and proteins FOXM1 and p27^Kip1^ are expressed during embryonic and postnatal stages in the mouse cochlea [8]. Moreover, neurosensory cells of the organ of Corti are highly dependent on p27^Kip1^ expression to maintain their proliferative quiescence, even after their developmental exit from the cell cycle [61].

## 3. Animal Models of Hearing Loss Associated with Human Mutations in the *IGF1* System

Pre-clinical models, in particular genetically-modified mice, have been instrumental in revealing the role of the IGF system in hearing and hearing loss. Gold-standard *in vivo* noninvasive methods for assessing the mouse auditory phenotype include the recording of the auditory brainstem response (ABR) to sound and the detection of the distortion product otoacoustic emissions (DPOAEs). ABR provides a reliable representation of hearing sensitivity (hearing thresholds), whereas DPOAEs inform about robustness of outer hair cells function [62]. In addition to this screening, susceptibility to auditory damage can be evaluated exposing mice to noxious agents like noise, ototoxic drugs or aging. In the following sections, we document findings from the most relevant mouse models with mutations in genes encoding for elements of the GH axis, the IGF system and downstream signaling pathways (Supplementary Table S1). 

### 3.1. GH System 

POU1F1 and PROP1 are transcription factors required for pituitary gland development. Spontaneous knockout mice for *Pou1f1* and *Prop1* (Snell and Ames dwarfs, respectively) present with a similar phenotype of pituitary hypoplasia and deficiency in the production of GH, prolactin and thyroid-stimulating hormone, but the impact on auditory function is quite different. *Pou1f1^dw^* mice show profound congenital deafness with significant outer hair cell loss and reduced endocochlear potential (the voltage in the endolymphatic spaces) [63]. Hearing deficits can be partially rescued by dietary thyroid hormone enrichment [64]; by contrast, *Prop1^df^* mice show delayed maturation of synaptic function and mildly reversible hearing loss [65]. GHRH binds to its specific receptor GHRHR to control GH synthesis and secretion in the pituitary gland. Patients with mutations in *GHRHR* [66,67] present with severe GH deficiency, mild high-tone sensorineural hearing loss (SNHL) and dizziness. Similar to the phenotype of *Pou1f1* and *Prop1* mutant mice, *Ghrh* and *Ghrhr* (Little) knockout mice show postnatal growth retardation, pituitary hypoplasia and decreased GH and IGF-1 levels, but curiously, they do not have an abnormal hearing phenotype [63] (IMPC database MGI:95709 and 95710, respectively). GH can be a potent neurotrophic factor for inner ear neurons [68], and addition of exogenous GH promotes hair cell regeneration in zebrafish [68]. Mice with targeted homozygous *Gh* mutations (e.g., *Gh^tm1.1(KOMP)Vlcg^*) exhibit dwarfism and pituitary hypoplasia, but no hearing deficit (IPMC database, MGI:95707), suggesting that GH is not essential for auditory function. Binding of GH to its receptor (GHR) initiates the JAK2 signaling cascade. Mutations in *GHR* cause Laron syndrome, a rare disease (ORPHA:633) characterized by marked short stature, low serum IGF-1 levels and auditory defects like late onset SNHL, auditory hypersensitivity and lack of acoustic stapedial reflexes, which may be prevented by IGF-1 treatment [69]. Knockout mice for *Ghr* are Laron syndrome models and exhibit retarded postnatal growth, dwarfism, decreased plasma IGF-1 levels and small pituitary glands, the auditory phenotype of these animals has not been studied despite the fact that studies with patients show a predisposition to suffer SNHL [70].

Inhibition of JAK2/STAT3 signaling has been reported to exert a protective effect against noise-induced hearing loss (NIHL) and cochlear damage [71]. However, the cochlear phenotype has not been evaluated in mice homozygous for a null mutation in *Jak2* as they display an embryonic lethal phenotype at mid-gestation. Similarly, mutations in *STAT5B* cause a Laron syndrome-like phenotype (OMIM 245590), but no hearing loss phenotype has been reported in *Stat5b* and *Stat5a* null mice (MGI:103035 and 103036).

### 3.2. IGF System

Mutations in the insulin gene (*INS*) are associated with permanent neonatal diabetes mellitus, a rare disease (ORPHA99885) characterized by chronic hyperglycemia due to severe non-autoimmune insulin deficiency. Moreover, patients with diabetes typically present a slow progressive, bilateral, high-frequency SNHL [72]. Unlike humans, mice and rats have two functional insulin genes: *Ins1* and *Ins2* [73]. The organ of Corti is a target tissue for insulin in the mouse, which shows expression of the high-affinity insulin receptor (IR) [74]. Insulin was shown to influence both protein and lipid metabolism in the inner ear [75], although the mechanisms underlying the association between diabetes and inner ear dysfunction are not yet known. A number of targeted knockout mice of *Ins1* and *Ins2* have been developed as models of diabetes type 1 and 2, or neonatal diabetes, and the majority present decreased insulin secretion and hyperglycemia. However, no auditory phenotype has been reported (MGI database, MGI:384434 and 3116137). Null mutants for *Insr* grow slowly and die by seven days of age, and in consequence, auditory tests cannot be performed. 

IGF-1 is essential for normal embryonic and postnatal inner ear development in mice. Accordingly, the total absence of IGF-1 observed in *Igf1^tm1Arge^* knockout mice impairs survival, differentiation and maturation of the cochlear ganglion cells and causes abnormal innervation of the sensory cells in the organ of Corti, which leads to congenital severe SNHL [10,76,77]. These phenotypes are similar to those observed in patients with homozygous *IGF1* gene deletion [78]. Heterozygous *Igf1^tm1Arge/+^* mice show a moderate reduction in cochlear ganglion volume and neuronal size [10], although no significant differences in click-ABR thresholds were observed [77]. Moreover, *Igf1* haploinsufficiency in *Igf1^tm1Arge/+^* mice accelerates ARHL and increases NIHL [14,79]. 

*Igf2* null mice present intrauterine growth retardation, although the postnatal growth is normal. No hearing phenotype has been described for *Igf2* knockout mice (MGI database, MGI:96434), though it could be expected from the cochlear expression pattern (Figure 1). A lack of phenotype could be possibly due to compensation by IGF-1 or other family members.

Relevant perinatal alterations in the inner ear have been found in *Igf1r^tm2.1Arge^* null mutant mice, including reduced proliferation of pro-sensory cells, delayed development of hair cells and support cells, and misorientation of hair cell stereociliary bundles [9]. However, these mice usually die at birth due to respiratory failure [80], and conditional mutants or heterozygous mice are needed to better explore the adult phenotype. For example, UBC-CreERT2; *Igf1r^(flox/flox)^* transgenic mice show alterations in mitochondria and redox homeostasis [81] and impaired neuron survival, although no hearing deficits have been described. *Igf1r^+/−^* mice present with a smaller size relative to wild-type mice, very low serum IGF-1 levels [82] and extended lifespan, although no hearing phenotype has been reported. In sharp contrast, *Igf2r*-null mice exhibit a fetal lethal overgrowth syndrome that could be partially rescued by crossing onto *Igf2^−/−^* background [83]. No hearing or vestibular phenotype has been so far reported to this mutation (MGI database, MGI:96435). 

Phenotyping of double knockouts of *Igf1/Igf2*, *Igf1/Igf1r* and *Igf2/Igf1r* suggested that IGF-2 acts through a receptor independent of IGF1R [84]. This unknown receptor was identified as the IR (reviewed in PMID: 11316729). 

Regarding IGFBPs genes knockout mice, only *Igfbp3* mutants have been characterized for auditory function, although no differences were found compared with wild-type controls (IMPC database, MGI:96438) (Supplementary Table S1). 

### 3.3. IGF-1 Signaling 

IRS1 and IRS2 integrate insulin and IGF-1 signaling. *Irs1^−/−^* mice are smaller than wild-type mice, show insulin resistance and have an extended lifespan. Spontaneous *Irs1^sml^* mutant mice do not present with evident inner ear defects, but ABR thresholds are significantly elevated [25]. *Irs2^−/−^* mice are also insulin-resistant, but unlike *Irs1^−/−^* counterparts, the mice develop diabetes and have a much shorter lifespan. Additionally, *Irs2^tm1Mfw^* null mice show hypoinnervation of the cochlear ganglion and aberrant stria vascularis, which leads to profound congenital hearing loss, a phenotype that is delayed in *Irs2^−/−^Ptpn1^−/−^* double knockout mice [24].

No hearing phenotype has yet been described for mice with knockout of genes encoding the numerous catalytic and regulatory subunits of PI3K (*Pik3ca, Pik3cb, Pik3cd, Pik3cg, Pik3c2b, Pik3c2g, Pik3r5, Pik3c2a, Pik3r1*). However, homozygous null mutants for *Akt1* have increased ABR thresholds and a higher sensitivity to noise cochlear damage compared with wild-type mice [85]. PTEN catalyzes the dephosphorylation of PIP_3_ to PIP_2_, blocking the AKT signaling pathway. PTEN has been demonstrated to regulate the proliferation of auditory progenitors, and conditional homozygous knockout mice display a delayed withdrawal of auditory progenitors from the cell cycle and consequently, supernumerary hair cells [86]. 

Mice homozygous for targeted, gene trap and N-ethyl-N-nitrosourea-induced *Mtor* mutations exhibit embryonic lethality and abnormal embryogenesis (MGI database, MGI:1928394), precluding the analysis of hearing. However, heterozygous *Mtor^tm1a(EUCOMM)Wtsi/+^* knockout mice show a normal startle response and do not have a clear auditory phenotype (IMPC database, MGI:1928394). mTORC1 is notably activated in the cochlear neurosensory epithelium during aging, and inhibition with rapamycin prevents ARHL [87]. Conditional knockout of regulatory-associated protein of mTOR (*Raptor*) and *Tsc1*, which are regulators of mTORC1 signaling, result in contrasting phenotypes. *Raptorc* knockout mice show delayed ARHL [87], whereas *Tsc1-cKO* cochleae show oxidative stress and impaired antioxidant defenses, leading to the early death of cochlear hair cells and accelerated hearing loss [87]. Thus, activated mTORC1 signaling is a critical cause of ARHL, suggesting that reduction of mTORC1 activity in cochlear hair cells may be a potential strategy to prevent ARHL [87]. Similarly, mTORC1 was shown to be hyperactivated in the cochlea in a rat model of gentamicin-induced ototoxicity, and induced the degeneration of spiral ganglion neurons, which could be reversed by co-treatment with rapamycin [57,88].

Blocking GSK3β with selective inhibitors attenuates the cisplatin-induced cytotoxicity of auditory cells both *in vivo* and *in vitro*, likely by enhancing autophagy [89,90]. However, no hearing or vestibular phenotype has been reported for *Gsk3b* knockout mice (MGI database, MGI:1861437). Cyclin D1 is involved in the cell cycle exit of sensory and supporting cells of the mammalian inner ear during embryogenesis [91], but the hearing phenotype has not been evaluated in knockout mice. 

SHC and GRB2 mediate IGF-1 post-receptor signaling by activating the ERK MAPK pathway. Mice homozygous for all three SHC isoforms die around E11.5 with cardiovascular defects, but mice homozygous for a targeted mutation in single isoforms show an extended life span and a reduced cellular sensitivity to oxidative stress, although no hearing loss phenotype has been reported (MGI database, MGI:98296, 106180 and 106179). *Grb2^tm1Paw^* knockout mice show abnormal otic vesicle development [92], with increased apoptosis, and die by E7.5. 

Impeding RAS signaling with the farnesyltransferase inhibitors B581 and FTI-277 reduces c-JUN activation in neonatal rat hair cells and protects against gentamycin-induced ototoxicity *in vitro* [93]. By contrast, mice homozygous for targeted null mutations in *Hras* are viable and fertile with no gross defects in neuronal development and no hearing deficits, although transgenic *Hras* mice show cochlear developmental abnormalities with indirect evidence of hearing loss [94].

Knockout mice for *Raf1* show embryonic growth retardation, placental anomalies and lung defects, resulting in mid-gestation or neonatal lethality depending on the genetic background (more severe in B6 than in 129/Sv * CD-1) [95] and, therefore, auditory phenotypes cannot be tested. On the Ola:MF1 background, however, mice survive and exhibit profound deafness with a strong decrease in the expression of the K^+^ channel Kir4.1. Moreover, heterozygous mice have an increased susceptibility to NIHL [40]. 

Homozygous inactivation of *Map2k1* (MEK1) leads to reduced embryo size and mid-gestational lethality (MGI database, MGI:1346866), whereas *Map2k2* (MEK2) knockout mice are viable, fertile, and apparently normal, with no significant hearing phenotype (IMPC database, MGI:1346867). Similarly, null mutants for *Mapk3* (ERK1) on a CD-1 background exhibit normal Mendelian ratios, growth, and show no obvious abnormalities (MGI:1346859). Conversely, *Mapk1* (ERK2) knockout mice die shortly after implantation. Conditional knockout mice deficient for *Erk2* in the inner ear hair cells (*Mapk1^tm1.2Kuta/tm1.2Kuta^ Tg(Nes-cre)1Kag/0)* have normal hearing, but are more vulnerable to noise [43]. 

## 4. GH-IGF-1 Axis Human Mutations Associated with Hearing Impairment

The GH-IGF-1 axis is an important regulator of human body growth and, accordingly, alterations of this axis commonly lead to short stature. Here, we have reviewed human GH/IGF1 system disorders associated to hearing loss (Table 1 and Supplementary Table S2).

### 4.1. IGF System and Signaling Mutations 

Homozygous human mutations in *IGF1* cause a rare disease (OMIM 608747) characterized by the association of intrauterine and postnatal growth retardation with intellectual deficit and sensorineural deafness (Table 1).

The first fully studied patient with this disease had a homozygous partial deletion of the *IGF1* exons 4 and 5, resulting in a truncated IGF-1 protein with only 25 of its 70 amino acids. The 15-year-old boy carrying the mutation presented with severe prenatal and postnatal growth failure, microcephaly, mental retardation, infertility and profound bilateral sensorineural deafness. Serum analysis revealed undetectable IGF-1 levels, elevated GH levels and normal values for IGFBP3 and ALS [78]. The second well-described case was a 55-year-old patient with a homozygous missense mutation in *IGF1* that changed the valine at position 92 to methionine (c.274G > A). Clinical features included severe growth retardation, delayed skeletal maturation, microcephaly, mental retardation and severe bilateral hearing loss. In contrast to the first case, the biochemical parameters were characterized by high levels of IGF-1 and an extremely low IGF-1 binding affinity for IGF1R [96]. More recently, a novel homozygous *IGF1* missense mutation has been described in a 3.2-year-old boy, changing a highly conserved tyrosine residue (Tyr60) to histidine (c.322T > C). Tyr60, located in the A domain of IGF-1 [34], has been described to be critical for the interaction with IGF1R. Next-generation sequencing analysis ruled out the presence of possible mutations in other genes of the IGF system such as *IGF1R*, *IGF2*, *IGF2R*, *IGFBP3* or *IGFALS*. Clinical features included intrauterine growth restriction, severe postnatal growth failure, mild developmental delay, microcephaly, facial dimorphism and bilateral sensorineural deafness. The patient had normal to mildly-elevated circulating IGF-1, variable basal levels of GH and normal-to-high IGFBP3 levels [97]. Although the clinical features described in the three patients are generally similar, the variability presented in the serum IGF-1 levels of the three mutations associated with sensorineural deafness is striking, particularly in the second and third cases, which respectively present very high or normal IGF-1 levels. These data point to the critical role of the effective binding of IGF-1 to its receptor.

**Table 1:** Human *IGF1* gene mutations associated with auditory functional studies.

	Woods et al., 1996 [78]	Walenkamp et al., 2005 [96]	Netchine et al., 2009 [98]	Keselman et al., 2019 [97]	Batey et al., 2014 [99]	Fuqua et al., 2012 [100]
**Mutation type**	Deletion Homozygous	Missense mutation Homozygous	Missense mutation Homozygous	Missense mutation Homozygous	Deletion Heterozygous	Splicing mutation Heterozygous
**Mutational analysis**	181 bp ex. 4–5	c.274G ˃ A, p.V92M ^(1)^	c.251G > A, p.R84Q ^(2)^	c.332T > C, p.Y108H	262 kb, entire *IGF-1* gene	Splicing excision ex 4 c.402+1G > C, p.N74Rfs*8
**Coordinates (GRCh38)**	Not mapped	chr12: 102419637	chr12: 102419660	chr12: 102419589	Not mapped	chr12: 102419508
**Age & clinical data**	♂ 15.8 yr Pre- and postnatal growth failure Microcephaly Micrognathia Clinodactyly Cognitive delay	♂ 55 yr Pre- and postnatal growth failure Microcephaly Dysmorphic features Severe cognitive delay Deaf-mutism	♂ 11 mo–9 yr Pre- and postnatal growth failure Microcephaly Non-dismorphic Clinodactyly Mild cognitive delay	♂ 3.2–7.8 yr Pre- and postnatal growth failure Microcephaly Dysmorphic features Hyperactive behaviour Developmental delay	♂ 2.3–8.4 yr Pre- and postnatal growth failure Microcephaly Micrognathia Clinodactyly Cognitive delay	♂ 8.8 yr Severe postnatal growth failure Normal physical examination Normal cognitive development
**Consanguinity**	Yes	Yes	Yes	Yes	No	No
**Birth weight (kg)**	1.4 (−3.9 SD)	1.4 (−3.9 SDS)	2.3 (−2.4 SDS)	1.9 (−3.1 SDS)	2.7 (−1.5 SDS)	3.0 (−1.5 SDS)
**Birth length (cm)**	37.8 (−5.4 SD)	39 (−4.3 SDS)	44 (−3.7 SDS)	38 (−6.3 SDS)	47.6 (−1.2 SDS)	47 (−0.6 SDS)
**Growth weight (kg)**	15.8 yr: 23 (−6.5 SD)	ND	11 mo: 5.3 (−5.0 SDS) 2.8 yr: 7.0 (−7.0 SDS)	3.2 yr: 6.1 (−5.1 SDS) 7.8 yr: 9.6 (−5.0 SDS)	2.3 yr: 8.8 (−3.8 SDS) 8.4 yr: 21.9 (−1.5 SDS)	8.8 yr: 21 (−2.1 SDS)
**Growth height (cm)**	15.8 yr: 119.1 (−6.9 SD)	55 yr: 117.8 (−8.5 SDS)	11 mo: 64 (−3.7 SDS) 2.8 yr: 76 (−4.9 SDS)	3.2 yr: 74 (−6.2 SDS) 7.8 yr: 90.2 (−6.5 SDS)	2.3 yr: 77.5 (−3.1 SDS) 8.4 yr: 114.9 (−2.7 SDS)	8.8 yr: 109 (−4.0 SDS)
**Auditory function**	Severe BHL (15.8 yr)	Severe BHL (55 yr)	Normal hearing (9 yr)	BHL (3.2 yr)	Normal hearing	Normal hearing
**IGF-1 levels (ng/mL)**	Undetectable 15 yr: <3	Very high 55 yr: 606 (+7.3 SDS)	Low 2.7 yr: 11 (before GH treatment)	Variable 4 yr: 47 (−1.15 SDS) 6.4 yr: 206 (+2.95 SDS)	Low-normal. 2.3 yr: 43.7; 5.3 yr: 58.5 8.4 yr: 100	Low-normal 9.3 yr: 115 (−2.2 SDS) (before GH treatment)
**IGFBP-3 levels (mg/L)**	Normal 15 yr: 3.3	Normal 55 yr: 1.98 (+0.1 SDS)	Normal-high (after GH treatment)	Normal-high (−1.58 to +2.31 SDS)	Normal 5.3 yr: 4.3; 8.4 yr: 5.4	Normal 9.3 yr: 2.4 (−1.2 SDS) (before GH)
**ALS levels (mg/L)**	Normal	High 55 yr: 28.9 (+3.4 SDS)	Normal-high (after GH treatment)	ND	Normal 5.3 yr: 10	Normal-high 9.3 yr: 13 (before GH)
**IGF-1 affinity for IGF1R**	None	Extremely low 90-fold lower	Partially reduced 3.9-fold lower	Reduced	ND	ND

Abbreviations: BHL, bilateral hearing loss; mo, month; ND, not determined; SD, standard deviation; SDS, standard deviation score; yr, year. ^(1)^ Previously described as p.V44M [96]. ^(2)^ Previously described as p.R36Q [98]. Source of information: The Human Gene Mutation Database (HGMD^®^) available at http://www.hgmd.cf.ac.uk/ac/index.php, accessed on 20 August 2021; Online Mendelian Inheritance in Man (OMIM) Catalogue available at https://www.omim.org/, accessed on 20 August 2021; PubMed Database available at https://pubmed.ncbi.nlm.nih.gov/, accessed on 20 August 2021. Table updated from Rodríguez-de la Rosa et al., 2017 [79].

A fourth homozygous mutation of *IGF1* has been reported, in which the patient did not present with sensorineural deafness [98]. The authors identified a homozygous missense *IGF1* mutation causing the replacement of a highly conserved arginine (Arg84) with a glutamine (c.251G > A) in the C domain of the protein. The patient showed intrauterine growth restriction, progressive postnatal growth failure, microcephaly, mild intellectual impairment and normal hearing. Serum IGF-1 levels in the patient at the age of 2.7 years were low and remained unchanged after GH treatment, in contrast to normal-high IGFBP3 and ALS levels. The mutated IGF-1 showed partially reduced affinity for IGF1R [98]. The partial IGF-1 deficiency produced marked effects on pre- and postnatal growth, brain development and cognitive functions. However, the development of the inner ear was apparently normal. There are no data on the follow-up of these patients, hence, the potential susceptibility to otic injury or accelerated hearing loss with aging is not known.

Heterozygous mutations in *IGF1* have also been described. The first case of a complete heterozygous *IGF1* deletion was reported in 2014, with a 262-kb deletion of chromosome 12 including the entire *IGF1* gene. Although the patient’s phenotype was milder than that reported for homozygous *IGF1* mutations, he had several common features including microcephaly and developmental delay. However, no cognitive delay or sensorineural deafness was observed. IGF-1 levels were in the low-normal range, with normal levels of IGFBP3 and ALS [99]. Two other heterozygous *IGF1* mutations have been published, occurring by insertion [101,102] and by splicing [100] mechanisms. Poor growth was a common clinical feature with normal hearing in the first case, whereas in the second case no functional studies were described. Both cases showed low IGF-1 levels and normal IGFBP3 and ALS levels, with no IGF-1 affinity for IGF1R in the splicing mutation.

Mutations are much more frequent in *IGF1R* than in *IGF1*. The Human Gene Mutation Database (HGMD^®^) lists 134 mutations in *IGF1R* and, according to a recent publication, 36 different mutations are pathogenic, and some are variants of uncertain significance [103]. Most of the mutations are heterozygous and only two homozygous *IGF1R* mutations have been described [104,105]. Patients with mutations in *IGF1R* show intrauterine growth retardation and postnatal growth failure, resulting in short stature and microcephaly (OMIM 270450). To our knowledge, hearing loss has been described in only two cases [106].

Mutations of the chromosomal region 11p15.5, which harbours the gene for IGF-2, are associated with Silver-Russell syndrome (OMIM 616489), an imprinting disorder characterized by severe pre- and postnatal growth restriction and dysmorphic facial features such as distinctive triangular face with prominent forehead and low-set ears [107,108,109,110]. A recent report described the first case of maternal uniparental disomy 7 leading to Silver-Russell syndrome and Pendred’s syndrome, but deafness was caused by a homozygous variant of *SLC26A4* (encoding Pendrin) [111].

The regulatory subunit of PI3K encoded by *PIK3R1* plays a crucial role in the activation of IGF-1, and heterozygous mutations in *PIK3R1* are responsible for SHORT syndrome (OMIM 269880), a rare condition with its main clinical features defined by its acronym: short stature (S), hyperextensibility of joints and/or hernias (H), ocular depression (O), Rieger anomaly (R) and teething delay (T). In addition to these classic features, hearing loss is also characteristic of patients with SHORT syndrome [112]. 

Heterozygous mutations in *MAPK1* are associated with Noonan syndrome (OMIM 619087), which is the most frequent neurodevelopmental disorder among the *RASopathies*, and is caused by pathogenic variants in at least ten genes encoding proteins with important roles in the MAPK signaling pathway. Noonan syndrome is characterized by developmental delay and impaired intellectual development of variable severity [113]. In 2015, van Trier and colleagues conducted a retrospective study in a cohort of 97 patients with Noonan syndrome and described external ear anomalies and hearing impairment. Hearing disorders in Noonan syndrome are associated with dysmorphic external ear morphology and both sensorineural and conductive hearing impairment. The authors studied the genotype-phenotype correlations for hearing loss and concluded that hearing loss is still an underestimated problem in this disease, considering that hearing impairment may progress during childhood or adulthood [114].

### 4.2. Human Hearing Loss Syndromes and Involvement of GH 

Syndromes with GH-associated hearing loss include both GH deficiency and GH resistance (Supplementary Table S2). In the case of decreased sensitivity to GH, hearing impairment results from a reduction in the levels of IGF-1 [115,116]. As mentioned previously in the mouse model discussion, homozygous or compound heterozygous mutations in *GHR* are responsible for GH insensitivity syndrome, also known as Laron syndrome (OMIM 262500). Laron syndrome is an autosomal-recessive disorder characterized by short stature. The disorder is caused by GHR dysfunction and a failure to generate IGF-1 in response to GH, whose levels can be normal or elevated [117]. Laron syndrome has also been associated with hearing impairment, and the only treatment available thus far is recombinant human IGF-1 (rhIGF-1) therapy. A prospective clinical study testing auditory function in patients with congenital IGF-1 deficiency in the early-treated, late-treated and untreated states revealed an association between Laron syndrome and SNHL, auditory hypersensitivity and lack of acoustic stapedial reflexes, and concluded that IGF-1 replacement therapy at early developmental age could prevent Laron syndrome [69]. GH deficiency is an endocrine disorder that leads to hearing impairment, abnormal pituitary development, and early growth failure and dysmorphic facial features. Hearing impairment includes both transient partial conductive hearing loss [118] and bilateral SNHL due to mutations in *POU3F4*, which cause X-linked sensorineural deafness DFN3 [119]. In 2017, Muus et al., performed an auditory study to evaluate the prevalence, type, and severity of hearing impairment in children with untreated GH deficiency, finding that 83% of young patients presented with conductive, mixed or SNHL with a predisposition to be bilateral [120,121].

Alström syndrome (OMIM 203800) is an autosomal-recessive disorder associated with impaired GH reserve [116,122], and is characterized by progressive cone-rod dystrophy, SNHL, insulin resistance with hyperinsulinemia, type 2 diabetes, cardiomyopathy, and progressive hepatic and renal failure [123,124]. Other syndromes presenting with SNHL but with normal GH levels include CHARGE syndrome (OMIM 214800) [125] and Turner syndrome (OMIM 163950) [116,126]. The molecular bases of the SNHL associated with Turner syndrome is not known, but the possible influence of oestrogen deficiency, cell cycle delay, IGF-1 deficiency and the histone demethylase gene *KDM6A* have been considered [126]. Finally, acromegaly is a chronic disorder caused by GH excess and is characterised by progressive somatic disfigurement and systemic manifestations such as hearing loss. Thus far, studies of acromegaly have associated this disorder indistinctly with conductive, sensorineural or mixed hearing loss, but with no consistent results on the cause-effect relationship between the disease and hearing loss [127].

### 4.3. GH-IGF-1 Axis and Therapeutic Potential

Different experimental and clinical approaches have recently demonstrated the key role of IGF-1 in the maintenance of auditory function [128]. As previously reported, circulating levels of IGF-1 stimulated by GH are related to the level of hearing loss presented by patients with *IGF1* mutations. Against this background, a multicenter and randomized clinical trial compared topical IGF-1 therapy and intratympanic corticosteroid therapy in patients with sudden sensorineural hearing loss (SSHL) refractory to systemic corticosteroids. The IGF-1 treatment showed promising results on hearing recovery in patients and a favourable safety profile for topical IGF-1 therapy [129]. In a similar context, recent clinical trials have demonstrated several side-effects of Teprotumumab, a human monoclonal antibody and IGF1R antagonist approved for the treatment of thyroid eye disease in adults. Hearing loss was reported in eight patients in the treatment group versus 0 in the placebo group. Hearing impairment included unilateral and bilateral symptoms of hearing loss and tinnitus [130]. Moreover, in a recent study characterizing the cochlear microenvironment by measuring different inflammatory markers in perilymph samples of patients receiving cochlear implantation, higher levels of IGFBP1, a key regulator of IGF-1, were measured in patients with complete loss of auditory function compared with patients with residual hearing [131]. rhIGF-1 has been approved as an orphan drug for the treatment of growth failure in children and adolescents with severe primary IGF-1 deficiency including Laron syndrome. To improve the available pharmacokinetic data regarding this treatment, a recent study undertook a safety and efficacy analysis of rhIGF-1 replacement therapy through the sequential measurements of serum IGF-1, glucose, potassium, insulin and cortisol in patients. Results revealed an inverse correlation between IGF-1 and serum potassium concentrations after injections of rhIGF-1, whereas no significant correlation was detected between serum IGF-1 concentrations and insulin or glucose concentrations [132].

With the exception of a recent case report that showed for the first time the recovery of hearing loss after GH administration in a child diagnosed with cerebral palsy and bilateral SNHL [133], the efficacy of GH treatment on hearing has not been sufficiently demonstrated. Because elevated levels of GH induce greater ossification of the ear cavities, it might be thought that this would trigger a worsening of auditory transmission, as apparently observed in acromegaly [127]. However, GH therapy does not appear to increase the risk of hearing loss in patients with Turner syndrome as concluded in different studies [134,135]. A study of audiological outcomes in 173 children with GH deficiency and hearing impairment showed that hearing loss, with a predisposition to be bilateral, was prevalent in these patients, and mixed hearing loss was particularly common [120].

To sum up, here we review data showing that the expression of the IGF system components and IGF-1 intracellular targets is tightly regulated with specific developmental and cell-type patterns. IGF-1, its receptor, and downstream targets, participate in the regulation of multiple developmental processes, and are essential for the postnatal differentiation and myelination of the spiral ganglion neurons and scala media cell types and structures. In the adult cochlea, IGF-1 seems to be essential for cochlear metabolic homeostasis, as its deficiency–either total or partial– cause redox stress and chronic inflammation. Downstream of IGF-1, deleterious mutations in the genes coding for IRS, C-RAF, p38α, DUSP1 and AKT show strong hearing loss phenotypes in the mouse. These results provide the molecular and genetic bases to understand a battery of rare human deafness syndromes, as well as the molecular aspects of noise-induced and age-induced hearing loss. Furthermore, they provide a framework to better understand the beneficial effects of IGF-1 treatment for hearing loss and suggest that further exploration of downstream IGF-1 targets could both uncover new human deafness genes and foster the development of novel therapeutic strategies to prevent and cure hearing loss.

## Supplementary Materials

The following are available online at https://www.mdpi.com/article/10.3390/genes12101553/s1, Table S1: Hearing phenotype of IGF-1-related genetically modified mice, Table S2: Clinical conditions with GH/IGF-1 axis alterations.
