# Supplementary material for: Insulin-like Growth Factor 1 Signaling in Mammalian Hearing
Source: Genes (Basel). 2021 Sep 29;12(10):1553. doi: 10.3390/genes12101553 (PMC8535591; doi:10.3390/genes12101553)
Supplement: Supplementary file 1 [file genes-12-01553-s001.zip › genes-13605331_Table S1.pdf]

	Allelic composition	Phenotype
Pituitary development	$Pou1f1^{dw/dw}$ (*Snell dwarf*)	Decreased body size; extended life span; dwarfism; sterility; pituitary hypoplasia; GH, PL and TSH deficiency; enhanced insulin sensitivity; lower tumor incidence. MGI database, MGI:3653204.<br>Profound congenital deafness (~65 dB elevation) that can be rescued by oral TH replacement; reduced endocochlear potentials, cochlear microphonics and DPOAEs; tectorial membrane abnormalities (prominent Hensen's stripe, elevated β-tectorin composition and disrupted striated-sheet matrix); impaired expression of KCNJ10 in the stria vascularis; significant OHC loss [1,2].
	$Prop1^{df/df}$ (*Ames dwarf*)	Small adenohypophysis; lack of somatotropic and thyrotropic hormone producing cells; decreased GH and TSH hormone levels; small thyroid gland; proportional dwarf; model of hypopituitarism (ORPHA:95494). MGI database, MGI:3042606.<br>Mild hearing deficit (~15 dB elevation) at 6 week and normal hearing by 12 week; delayed development of OHC synaptic function; persistent abnormal expression of otoferlin in apical OHC [3].
GH release and signaling	$Ghrh^{tm1Salv/tm1Salv}$	Pituitary hypoplasia and decreased GH levels; postnatal growth retardation and decreased body weight; reduced IGF-1 circulating levels. Model of isolated growth hormone deficiency type IA (ORPHA:231671). MGI database, MGI:3051589.<br>No significant phenotype detected. IMPC database, MGI:95709.
	$Ghrhr^{tm1.1(KOMP)Vlc/tm1.1(KOMP)Vlcg}$	Increased total body fat amount and decreased lean body mass; decreased heart weight; decreased bone mineral density; thrombocytopenia; decreased glucose and increased triglyceride circulating levels; poorer grip strength; abnormal sleep behavior.<br>Abnormal ABR wave morphology, but thresholds are not increased. IMPC database, MGI:95710.
	$Gh^{tm1.1(KOMP)Vlcg/tm1.1(KOMP)Vlcg}$	Decreased body length; decreased spleen, liver, kidney and brain weight; small heart; decreased grip strength and hypoactivity; impaired glucose tolerance.<br>No significant phenotype detected. IMPC database, MGI:95707.
	$Ghr^{tm1Arge/tm1Arge}$<br>$Ghr^{tm1Jjk/tm1Jjk}$<br>$Ghr^{tm1.1Jero/tm1.1Jero}$<br>$Ghr^{tm1.2Masp/tm1.2Masp}$<br>$Ghr^{tm1Mwat/tm1Mwat}$<br>$Ghr^{tm2Mwat/tm2Mwat}$	Postnatal growth retardation and proportional dwarfism; decreased IGF-1 and increased GH circulating levels; reduced fertility. $Ghr^{tm1Arge/tm1Arge}$ and $Ghr^{tm1Jjk/tm1Jjk}$ are considered models of Laron syndrome (ORPHA:633). MGI database, MGI:2681520 and MGI:3796418, respectively.
Factors	$Ins1^{em1(IMPC)Marc/em1(IMPC)Marc}$<br>$Ins1^{tm1Jja/tm1Jja}$<br>$Ins2^{tm1Jja/tm1Jja}$	

Table S1. Hearing phenotype of IGF-1-related genetically modified mice. Non-exhaustive list of genetically modified mice with mutations in the main components of the GH axis, IGF system and IGF1 signaling pathway, which have shown an auditory phenotype. Source of information: Mouse Genome Informatics database, available at http://www.informatics.jax.org/ ; International Mouse Phenotype Consortium, available at https://www.mousephenotype.org/ ; Disease Ontology database, available at https://disease-ontology.org/; Pubmed NH database, available at https://pubmed.ncbi.nlm.nih.gov/ . *Pou1f1*, POU domain class 1 transcription factor 1; *Prop1*, PROP paired-like homeobox domain 1; *Ghrh*, Growth hormone-releasing hormone; *Ghrhr*, Growth hormone-releasing hormone receptor; *Gh*, Growth hormone ; *Ghr*, Growth hormone receptor; *Ins1/2*, Insulin 1/2; *Igf1/2*, Insulin-like growth factor 1/2; *Insr1/2*, Insulin receptor 1/2; *Igf1r/2r*, Insulin-like growth factor 1/2 receptor; *Igfbp*, Igf binding proteins; *Irs1/2*, Insulin receptor substrate 1/2; *Pik3r/c*, Phosphatidylinositol-4,5-bisphosphate 3-kinase catalytic/regulatory subunit; *Pdpk1*, 3-phosphoinositide dependent protein kinase 1; *Mtor*, Mechanistic target of rapamycin kinase; *Akt*, AKT serine/threonine kinase; *Tsc*, Tuberous sclerosis; *Atoh1*, Atonal bHLH

	Igf1$^{tm1Arge/tm1Arge}$	Postnatal lethality and growth retardation; decreased body weight; disproportionate dwarf; delayed bone ossification; abnormal lung morphology; increased neuron apoptosis; infertility; decreased circulating IGF-1 levels.<br>Severe cochlear abnormalities (small cochlea, thicker cartilaginous otic capsule, delayed tectorial membrane maturation, cochlea and cochlear ganglion volumes reduced, reduced average size and altered myelin sheath of cochlear ganglion neurons, increased apoptosis and altered expression of neurofilament and vimentin in cochlear neurons) [4,5]; prematurely aged stria vascularis reminiscent of the diabetic strial phenotype [6]; severe hearing impairment (all-frequency involved bilateral sensorineural syndromic HL, abnormal ABR waveform shape, increased click-ABR thresholds, increased click-ABR latencies and interpeak latencies, delayed transmission in the central auditory pathway, increased wave II amplitude) [7]; cochlear nucleus alterations (decreased area and cell size, cell loss; upregulation of VGluT1, but not VGAT, downregulation of MEF2 transcription factor, increased efficacy of glutamatergic synapses) [8].
	Igf1$^{tm1Arge/+}$	40% less expression of *Igf1* transcript; low IGF-1 serum levels.<br>At P20 (but not P5), heterozygotes display a moderate reduction in cochlear ganglion volume (10%) and in neuronal size (12%) relative to wild-type mice [4] ; at 3 months, heterozygotes display no significant differences in click-ABR thresholds and interpeak I-IV latencies relative to wild-type mice [7]; greater susceptibility to NIHL; chronic subclinical proinflammatory age-associated state, with increased expression of inflammatory genes coding for cytokines TGF-β1 and IL-1β in the cochlea [9] .
	Igf1$^{tm2Arge/tm2Arge}$<br>*Igf1*$^{tm2.1Arge/tm2.1Arge}$<br>*Igf1*$^{tm1.1Dlr/tm1.1Dlr}$<br>*Igf1*$^{tm1.1Nros/tm1.1Nros}$<br>*Igf1*$^{tm1Ts/tm1Ts}$<br>*Igf1*$^{tm2Ts/tm2Ts}$	These *Igf1* mutants show some of the following symptoms: delayed embryonic growth; postnatal lethality and growth retardation; decreased body weight; infertility; decreased circulating IGF-1 levels; increased or decreased circulating GH levels; insensitivity to GH; decreased circulating glucose levels; decreased long bone epiphyseal plate size; abnormal respiratory morphology and physiology; abnormal brain morphology and physiology; abnormal muscle morphology; increased ventricle muscle contractility and systemic arterial blood pressure. MGI database, MGI:96432.
	Igf2$^{tm1Rob/tm1Rob}$	Decreased birth weight; small placenta. Heterozygous *Igf2*$^{tm1Snha/+}$, *Igf2*$^{tm1Wrk/+}$, *Igf2*$^{tm2Wrk/+}$, *Igf2*$^{tm3Wrk/+}$ and *Igf2*$^{tm4Wrk/+}$ present embryonic and postnatal growth retardation; decreased birth weight; abnormal postnatal growth; abnormal skeleton morphology, with thinner and shorter bones. MGI database, MGI:96434.
Receptors	*Insr*$^{tm1Dac/tm1Dac}$<br>*Insr*$^{tm1Jja/tm1Jja}$<br>*Insr*$^{M2Btlr/M2Btlr}$<br>*Insr*$^{tm1Shs/tm1Shs}$	Postnatal lethality; decreased body weight; increased circulating glucose and insulin levels; hepatic steatosis. MGI database, MGI:96575.
	Igf1r$^{tm2.1Arge/tm2.1Arge}$	Prenatal growth retardation; neonatal lethality due to respiratory failure. MGI database, MGI:5306047.<br>Shortened and slightly widened cochlea; smaller and flattened bony labyrinth; shorter cochlear sensory epithelium; delayed development of OHC, IHC and support cells [10].
	Igf2r$^{tm1.1(IGF2R)Arte/tm1.1(IGF2R)Arte}$<br>*Igf2r*$^{tm1Arge/tm1Arge}$<br>*Igf2r*$^{tm2Arge/tm2Arge}$	Perinatal and postnatal lethality; elevated IGF-2 serum levels; enlarged heart with dilated ventricles; congestive heart failure; enlarged lung, kidney and liver; polydactilia; kinked tail; reduced fertility. MGI database, MGI:96435.

	Igf2r$^{tm1Stw/tm1Stw}$<br>*Igf2r*$^{tm1Wag/tm1Wag}$	
Binding proteins	*Igfbp1*$^{tm1Taub/tm1Taub}$<br>*Igfbp2*$^{tm1Jep/tm1Jep}$<br>*Igfbp3*$^{tm1b(KOMP)Wtsi/tm1b(KOMP)Wtsi}$<br>*Igfbp3*$^{tm1Jep/tm1Jep}$<br>*Igfbp3*$^{tm1Lex/tm1Lex}$<br>*Igfbp4*$^{tm1Jep/tm1Jep}$<br>*Igfbp5*$^{tm1Jep/tm1Jep}$	These mutants show some of the following symptoms: increased liver weight; liver inflammation; increased hepatocyte apoptosis; hepatic steatosis and necrosis; decreased heart and kidney weight; small spleen; abnormal retinal vasculature morphology; retinal oxidative stress; increased circulating glucose and insulin levels; decreased body weight; impaired glucose tolerance.<br>No significant phenotype detected in the *Igfbp3*$^{tm1b(KOMP)Wtsi}$ mice. IMPC database, MGI:96572.
Insulin receptor substrate	*Irs1*$^{Sml/Sml}$ *(Small)*	Decreased body weight; proportional dwarf; decreased percent body fat; decreased circulating glucose levels; increased circulating insulin levels and insulin resistance; abnormal bone structure and mineralization; short and thin tail. MGI database, MGI:4829466.<br>Significantly all-frequencies higher thresholds than control littermates; no obvious defects in the inner ear histology [11].
	Irs1$^{tm1Jos/tm1Jos}$<br>*Irs1*$^{tm1Tka/tm1Tka}$<br>*Irs1*$^{tm2.1Mfw/tm2.1Mfw}$<br>*Irs1*$^{tm3.1Mfw/tm3.1Mfw}$	These mutants show some of the following symptoms: decreased body weight; postnatal growth retardation; decreased bone mineral density; increased circulating insulin levels; insulin resistance; impaired glucose tolerance; pre-weaning lethality. MGI database, MGI:99454.
	Irs2$^{tm1Mfw/tm1Mfw}$	Decreased body weight; premature death; decreased pancreatic beta cell mass; abnormal glucose homeostasis; polydipsia, polyuria and polyphagia; small pituitary gland; reduced fertility; model of type 2 diabetes mellitus. MGI database, MGI:3583763.<br>Profound sensorineural deafness; elevated hearing thresholds in response to click and pure tone stimuli, high frequencies (28 and 40 kHz) being most affected; increased latencies and deceased peak I amplitude; strial atrophy with marginal cell degeneration, dilatation or merging of the capillaries and altered Kir4.1 expression; significant reduction in myelin P0 immunoreactivity in the cochlear ganglion and fibers projecting to the sensory cells [12]. Double knock out mice for IRS2 and PTP1B coding genes show normal hearing phenotype [12].
	Irs2$^{tm1Tka/tm1Tka}$	Decreased pancreatic beta cell mass; increased insulin secretion and insulin levels; hyperglycemia; increased circulating cholesterol, fatty acids and triglycerides levels; increased systemic arterial systolic blood pressure. Model of type 2 diabetes mellitus. MGI database, MGI:3583344.

transcription factor 1; *Rheb*, Ras homolog MTORC1 binding; *Gsk3b*, Glycogen synthase kinase 3 beta; *Gys1*, Glycogen synthase 1; *Ccnd1*, Cyclin 1; *Shc1*, SHC transforming protein 1; *Grb2*, Growth factor receptor-bound protein 2; *Sos1*, SOS Ras/Rac guanine nucleotide exchange factor 1; *Hras*, HRAS protooncogene; *Raf1*, RAF1 protooncogene serine/threonine kinase; *Rasgrf*, Ras protein specific guanine nucleotide releasing factor 1; *Mapk*, Mitogen-activated protein kinase; *Dusp1*, Dual-specificity phosphatase 1. Abbreviations: ABR, auditory brainstem response; DPOAE, distorsion product of otoacoustic emissions; GH, growth hormone; IHC, inner hair cell; HC, hair cell; HL, hearing loss; NIHL, noise induced hearing loss; OHC, outer hair cell; PL, prolactin; TH, thyroid hormone; TSH, thyroid-stimulating hormone.

References

PI3K subunits	*Pik3ca*$^{tm1Nbm/tm1Nbm}$<br>*Pik3cb*$^{tm1.1Bvan/tm1.1Bvan}$<br>*Pik3cb*$^{tm1a(EUCOMM)Wts/tm1a(EUCOMM)Wtsi}$<br>*Pik3cd*$^{tm1Tnr/tm1Tnr}$<br>*Pik3cg*$^{tm1Dwu/tm1Dwu}$<br>*Pik3cg*$^{tm1Ehi/tm1Ehi}$<br>*Pik3c2a*$^{tm1b(EUCOMM)Hmgu/tm1b(EUCOMM)Hmgu}$<br>*Pik3c2g*$^{tm1Ehi/tm1Ehi}$<br>*Pik3r1*$^{tm1Dfr/tm1Dfr}$<br>*Pik3r5*$^{tm1Lste/tm1Lste}$	*Pik3c* mutants show some of the following symptoms: embryonic growth retardation; embryonic or pre-weaning lethality; increased circulating glucose, insulin, cholesterol and triglyceride levels; impaired glucose tolerance; insulin resistance; decreased liver glycogen levels; decreased liver weight; hepatic steatosis abnormal heart ventricle morphology. *Pik3r* mutants present postnatal lethality; decreased circulating insulin levels; hypoglycemia; hepatic necrosis; dystrophic cardiac calcinosis; abnormal brown adipose necrosis. MGI database, MGI:1353576.
Akt and AKT quinases	*Pdpk1*$^{tm1.2Mlw/tm1.2Mlw}$<br>*Pdpk1*$^{tm1Bcol/tm1Bcol}$<br>*Pdpk1*$^{tm1Dral/tm1Dral}$<br>*Pdpk1*$^{tm1Ejm/tm1Ejm}$<br>*Pdpk1*$^{tm2Ejm/tm2Ejm}$	Mutants show some of the following symptoms: decreased embryo size and body weight; embryonic or perinatal lethality; abnormal craniofacial morphology; pancreatic islet hypoplasia; increased circulating insulin levels; insulin resistance; abnormal brain development. MGI database, MGI: 1338068.
	Mtor$^{flat/flat}$ *(Flat-top)*<br>*Mtor*$^{Gt(OST92090)Lex/Gt(OST92090)Lex}$<br>*Mtor*$^{tm1.1Clyn/tm1.1Clyn}$<br>*Mtor*$^{tm1.1Clyn/+}$<br>*Mtor*$^{tm1.1Koz/tm1.1Koz}$<br>*Mtor*$^{tm1.2Seq/tm1.2Seq}$<br>*Mtor*$^{tm1Yam/tm1Yam}$	*Mtor* mutants show: embryonic growth retardation and lethality; decreased body size; decreased circulating IGF-1 levels; increased total body fat amount and decreased lean body mass. MGI database, MGI: 1928394.
	Akt1$^{tm1Mbb/tm1Mbb}$	Increased sensitivity to noise injury [13].
	Akt1$^{tm1Hem/tm1Hem}$<br>*Akt2*$^{tm1Hem/tm1Hem}$/<br>*Akt3*$^{tm1Hem/tm1Hem}$	High ABR thresholds in single *Akt1*$^{-/-}$ knockout and *Akt2*$^{-/-}$*Akt3*$^{-/-}$ double knockout mice [14].
AKT dowstream signaling	*Tsc1*$^{tm1.1Djk/tm1.1Djk}$<br>*Tsc1*$^{tm1Chdl/tm1Chdl}$<br>*Tsc1*$^{tm1Hin/tm1Hin}$<br>*Tsc2*$^{tm1.2Mjg/tm1.2Mjg}$<br>*Tsc2*$^{tm1Djk/tm1Djk}$<br>*Tsc2*$^{tm1Tno/tm1Tno}$	*Tsc1* and Tsc2 knockouts: embryonic lethality during organogenesis; decreased embryo size; abnormal fetal cardiomyocyte morphology. Model of tuberous sclerosis (ORPHA:805). MGI data base, MGI:1929183 and 102548.<br>*Tsc1*$^{fl/fl}$*Atoh1-Cre*: no differences in hearing thresholds and cochlear morphology (sensory epithelia structure, OHC bundles pattern, expression of prestin, Sox2 and neurofilament markers), and functional mechanotransduction compared to controls; early-onset death of cochlear HCs and accelerated HL; oxidative stress and impaired antioxidant cochlear defenses; treatment with rapamycin and *N*-acetylcysteine rescued *Tsc1-cKO* HC from injury *in vivo* [15].

	$Tsc1^{fl/fl}$ *Atoh1-Cre*	
	$Rheb^{tm1.1Pfw/tm1.1Pfw}$<br>$Rheb^{tm1.1Yelg/tm1.1Yelg}$	Embryonic growth retardation; decreased embryo size; embryonic lethality during organogenesis; abnormal heart development; thin ventricular wall. MGI database, MGI:97912.
	$Gsk3b^{tm1.2Ypc/tm1.2Ypc}$<br>$Gsk3b^{tm1Dgen/tm1Dgen}$<br>$Gsk3b^{tm1Grc/tm1Grc}$<br>$Gsk3b^{tm1Jrw/tm1Jrw}$	*Gsk3b* mutants present: neonatal lethality or pre-weaning lethality; cleft palate; delayed bone ossification; abnormal liver morphology. MGI database, MGI:1861437.
	$Gys1^{Gt(OST33395)Lex/Gt(OST33395)Lex}$<br>$Gys1^{tm1.1Arte/tm1.1Arte}$<br>$Gys1^{tm1a(EUCOMM)Wtsi/tm1a(EUCOMM)Wtsi}$	Neonatal lethality; increased heart weight; pulmonary vascular congestion; abnormal glycogen homeostasis; decreased skeletal muscle glycogen levels. MGI database, MGI:101805.
	$Ccnd1^{tm1(CCNE)Pisc/tm1(CCNE)Pisc}$<br>$Ccnd1^{tm1Dsn/tm1Dsn}$<br>$Ccnd1^{tm1Phin/tm1Phin}$<br>$Ccnd1^{tm1Wbg/tm1Wbg}$<br>$Ccnd1^{tm2(Ccnd2)Pisc/tm2(Ccnd2)Pisc}$	Prenatal and postnatal lethality; postnatal growth retardation; decreased body weight; abnormal retina morphology and electrophysiology; abnormal mammary gland growth during pregnancy; limb grasping. MGI database, MGI:88313.
SHC-GRB2-SOS complex	$Shc1^{tm1Pgp/tm1Pgp}$	Extended life span; decreased susceptibility to atherosclerosis and to oxidative stress.<br>Decreased susceptibility to acoustic trauma; preserved auditory function and lower levels of oxidative stress and ischemia markers; attenuated age-related hearing loss and inner ear senescence [16].
	$Shc1^{tm1Lex/tm1Lex}$<br>$Shc1^{tm1Paw/tm1Paw}$<br>$Shc1^{tm8Paw/tm9.1Paw}$<br>$Shc2^{tm1Paw/tm1Paw}$	Embryonic or pre-weaning lethality; abnormal blood vessel morphology; thin myocardium and decreased cardiac muscle contractility; abnormal sensory neuron morphology; small dorsal root ganglion. MGI database, MGI:98296 and 106180.
	$Grb2^{tm1Paw/tm1Paw}$<br>$Grb2^{tm2Paw/tm2Paw}$<br>$Grb2^{tm1Paw/tm2Paw}$	Embryonic or neonatal lethality; abnormal heart development; abnormal facial and vagus nerve morphology.<br>$Grb2^{tm1Paw/tm2Paw}$ presents an abnormal otic vesicle development; increased mesenchymal cells apoptosis in the otic vesicle [17].
	$Sos1^{tm1.2Rak/tm1.2Rak}$<br>$Sos1^{tm1Dlb/tm1Dlb}$<br>$Sos1^{tm1Lowy/tm1Lowy}$	Embryonic lethality; decreased body weight; abnormal cardiovascular system (heart mispositioned or misorientated, enlarged heart, distended pericardium); abnormal craniofacial morphology; ocular hypertelorism. Model of Noonan syndrome 4 (ORPHA:648). MGI data base, MGI:5000309.

1. Mustapha, M.; Fang, Q.; Gong, T.-W.; Dolan, D.F.; Raphael, Y.; Camper, S.A.; Duncan, R.K. Deafness and Permanently Reduced Potassium Channel Gene Expression and Function in Hypothyroid Pit1dw Mutants. *J Neurosci* **2009**, *29*, 1212–1223, doi:10.1523/JNEUROSCI.4957-08.2009.

RAS-RAF pathway	*Hras*$^{tm2Xbr/tm2Xbr}$	Heart ventricle hypertrophy; enlarged heart atrium; abnormal cranium morphology; hypertension; renal fibrosis. Model of Costello syndrome (ORPHA:3071). MGI database, MGI:3805200.
	*Tg(Tyr-HRAS *G12V)#Acc/0*	Decreased body size; diluted coat color but hyperpigmentation of ears, neck, feet, and tail; abnormal brain meninges morphology; circling behavior; abnormal eye anterior chamber morphology and cataracts.<br>Cochlear space contains an abnormal tissue mass composed of poorly differentiated spindle-to stellate cells in a loose myxomatous-like arrangement and pigment-producing cells; mice show hearing alterations not confirmed by ABR [18].
	Raf1$^{tm1b(EUCOMM)Wtsi/tm1b(EUCOMM)Wtsi}$<br>*Raf1*$^{tm1Bacc/tm1Bacc}$<br>*Raf1*$^{tm1Ulb/tm1Ulb}$<br>*Raf1*$^{tm1.1Bgn/+}$	Embryonic growth retardation and lethality; prenatal lethality; decreased body weight; abnormal vasculogenesis and hemorrhages; liver hypoplasia; anemia. *Raf1*$^{tm1.1Bgn/+}$ presents cardiac hypertrophy; enlarged spleen; abnormal facial morphology; ocular hypertelorism. Model of Noonan syndrome 5 (ORPHA:648).
	Raf1$^{tm1Zim/tm1Zim}$	Lethality depending on the genetic background. Severe phenotype in a 129/Sv * CD-1 background with: embryonic growth retardation; embryonic lethality during organogenesis; decreased fetal weight; abnormal placenta morphology; neonatal lethality; small lung with abnormal pulmonary alveolus morphology; respiratory failure; abnormal retinal, epidermal and dermal layer morphology; underdeveloped hair follicles . MGI database: MGI:2429683 [19].<br>On OlaMF1 background, profound deafness but normal response to standard vestibular screening tests; loss of the K^+ channel Kir4.1 expression; increased susceptibility to noise injury in *C-Raf* heterozygous adult mice [20].
	Rasgrf1$^{tm1Esn/tm1Esn}$<br>*Rasgrf1*$^{tm1Kln/tm1Kln}$<br>*Rasgrf1*$^{tm1Sva/tm1Sva}$<br>*Rasgrf1*$^{tm1Toc/tm1Toc}$<br>*Rasgrf1*$^{tm1Toc/+}$<br>*Rasgrf2*$^{tm1Esn/tm1Esn}$	*Rasgrf1* mutants show decreased body weight; decreased pancreatic B-cell number; decreased insulin levels; impaired glucose tolerance; impaired contextual conditioning behavior and long term spatial reference memory; abnormal retina; heterozygous mice show abnormal pituitary secretion, with decreased levels of GH and IGF-1. *Rasgrf2* mutants present immune system abnormalities.<br>*Rasgrf1*$^{tm1Esn/tm1Esn}$: no differences in ABR parameters compared to wild type [21].
MEK-ERK	*Map2k1*$^{tm1.1Bacc/tm1.1Bacc}$<br>*Map2k1*$^{tm1.1Chrn/tm1.1Chrn}$<br>*Map2k1*$^{tm2.1Chrn/tm2.1Chrn}$<br>*Map2k2*$^{tm1Chrn/tm1Chrn}$	*Map2k1* (MEK1) mutants: embryonic lethality during organogenesis; increased cranium length; pulmonary artery stenosis. Model of cardiofaciocutaneous syndrome (ORPHA:1340). *Map2k2* (MEK2) no abnormal phenotype detected, apparently function can be compensated for by *Map2k1*. MGI database, MGI:1346866.
	Mapk3$^{tm1Gpg/tm1Gpg}$	Premature death; cardiac hypertrophy; decreased T cell proliferation. MGI database, MGI:1346859.
	Mapk1$^{tm1.2Kuta/tm1.2Kuta}$<br>*Tg(Nes-cre)1 Kag*	Abnormal response to novel objects; impaired cued conditioning behavior; abnormal maternal nurturing; astrocytosis; model of autism spectrum disorder.<br>Increased susceptibility to NIHL; significantly lower survival of IHC and poor recovery from noise-induced HL [22].

2. Karolyi, I.J.; Dootz, G.A.; Halsey, K.; Beyer, L.; Probst, F.J.; Johnson, K.R.; Parlow, A.F.; Raphael, Y.; Dolan, D.F.; Camper, S.A. Dietary Thyroid Hormone Replacement Ameliorates Hearing Deficits in Hypothyroid Mice. *Mamm Genome* **2007**, *18*, 596–608, doi:10.1007/s00335-007-9038-0.

Stress kinases and phospatases	$Mapk14^{tm1.1Lex/tm1.1Lex}$<br>$Mapk14^{tm1.1Otsu/tm1.1Otsu}$<br>$Mapk14^{tm1Gab/tm1Gab}$<br>$Mapk14^{tm1Jda/tm1Jda}$<br>$Mapk14^{tm1Mka/tm1Mka}$	Embryonic or perinatal lethal, showing multiple organ system defects; abnormal myoblast differentiation and delayed myofiber growth and maturation. MGI database, MGI:1346865.
	$Mapk14^{tm2Nbr/tm2Nbr}$<br>$Polr2a^{tm1(cre/ERT2)Bb/+}$	Abnormal lung morphology with multicellular septa and reduction of the alveolar lumen 2 weeks after tamoxifen treatment.<br>Decreased susceptibility to noise-induced HL; reduced ABR threshold shifts [23,24].
	$Dusp1^{tm1Bv/tm1Brv}$	Increased susceptibility to endotoxin shock; increased circulating levels of IL-10, IL-6 and TNFα; decreased body weight; decreased total body fat amount; decreased liver weight.<br>Premature progressive hearing loss, correlated with HC death, degeneration of spiral neurons and increased macrophage infiltration; elevated ABR thresholds from the age of 2 months; significant delay on wave I latency; DPOAE I/O functions significantly lower; loss of HC and supporting cells, loss of neural cells of the basal spiral ganglion and loss of spiral ligament fibrocytes, following a base to apex gradient that progressed with age; increased levels of reactive oxygen species and cochlear redox imbalance; inflammatory dysregulation, with up-regulation of pro-inflammatory cytokines *Il1b, Tnfa* and *Tgfb1* from 5 months [23]. Hearing loss phenotype is partially rescued by NAC-treatment from weaning [24].

3. Fang, Q.; Giordimaina, A.M.; Dolan, D.F.; Camper, S.A.; Mustapha, M. Genetic Background of Prop1(Df) Mutants Provides Remarkable Protection against Hypothyroidism-Induced Hearing Impairment. *J Assoc Res Otolaryngol* **2012**, *13*, 173–184, doi:10.1007/s10162-011-0302-3.

4. Camarero, G.; Avendano, C.; Fernandez-Moreno, C.; Villar, A.; Contreras, J.; de Pablo, F.; Pichel, J.G.; Varela-Nieto, I. Delayed Inner Ear Maturation and Neuronal Loss in Postnatal Igf-1-Deficient Mice. *J Neurosci* **2001**, *21*, 7630–7641.

5. Camarero, G.; Villar, M.A.; Contreras, J.; Fernández-Moreno, C.; Pichel, J.G.; Avendaño, C.; Varela-Nieto, I. Cochlear Abnormalities in Insulin-like Growth Factor-1 Mouse Mutants. *Hear Res* **2002**, *170*, 2–11, doi:10.1016/s0378-5955(02)00447-1.

6. Riquelme, R.; Cediel, R.; Contreras, J.; la Rosa Lourdes, R.; Murillo-Cuesta, S.; Hernandez-Sanchez, C.; Zubeldia, J.M.; Cerdan, S.; Varela-Nieto, I. A Comparative Study of Age-Related Hearing Loss in Wild Type and Insulin-like Growth Factor I Deficient Mice. *Front Neuroanat* **2010**, *4*, 27, doi:10.3389/fnana.2010.00027.

7. Cediel, R.; Riquelme, R.; Contreras, J.; Díaz, A.; Varela-Nieto, I. Sensorineural Hearing Loss in Insulin-like Growth Factor I-Null Mice: A New Model of Human Deafness. *Eur J Neurosci* **2006**, *23*, 587–590, doi:10.1111/j.1460-9568.2005.04584.x.

8. Fuentes-Santamaría, V.; Alvarado, J.C.; Rodríguez-de la Rosa, L.; Murillo-Cuesta, S.; Contreras, J.; Juiz, J.M.; Varela-Nieto, I. IGF-1 Deficiency Causes Atrophic Changes Associated with Upregulation of VGluT1 and Downregulation of MEF2 Transcription Factors in the Mouse Cochlear Nuclei. *Brain Struct Funct* **2016**, *221*, 709–734, doi:10.1007/s00429-014-0934-2.

9. Celaya, A.M.; Rodríguez-de la Rosa, L.; Bermúdez-Muñoz, J.M.; Zubeldia, J.M.; Romá-Mateo, C.; Avendaño, C.; Pallardó, F.V.; Varela-Nieto, I. IGF-1 Haploinsufficiency Causes Age-Related Chronic Cochlear Inflammation and Increases Noise-Induced Hearing Loss. *Cells* **2021**, *10*, 1686, doi:10.3390/cells10071686.

10. Okano, T.; Xuan, S.; Kelley, M.W. Insulin-like Growth Factor Signaling Regulates the Timing of Sensory Cell Differentiation in the Mouse Cochlea. *J Neurosci* **2011**, *31*, 18104–18118, doi:10.1523/JNEUROSCI.3619-11.2011.

11. DeMambro, V.E.; Kawai, M.; Clemens, T.L.; Fulzele, K.; Maynard, J.A.; Marín de Evsikova, C.; Johnson, K.R.; Canalis, E.; Beamer, W.G.; Rosen, C.J.; et al. A Novel Spontaneous Mutation of Irs1 in Mice Results in Hyperinsulinemia, Reduced Growth, Low Bone Mass and Impaired Adipogenesis. *J Endocrinol* **2010**, *204*, 241–253, doi:10.1677/JOE-09-0328.

12. Murillo-Cuesta, S.; Camarero, G.; González-Rodríguez, A.; De La Rosa, L.R.; Burks, D.J.; Avendaño, C.; Valverde, A.M.; Varela-Nieto, I. Insulin Receptor Substrate 2 (IRS2)-Deficient Mice Show Sensorineural Hearing Loss That Is Delayed by Concomitant Protein Tyrosine Phosphatase 1B (PTP1B) Loss of Function. *Mol Med* **2012**, *18*, 260–269, doi:10.2119/molmed.2011.00328.

13. Chen, J.; Yuan, H.; Talaska, A.E.; Hill, K.; Sha, S.-H. Increased Sensitivity to Noise-Induced Hearing Loss by Blockade of Endogenous PI3K/Akt Signaling. *J Assoc Res Otolaryngol* **2015**, *16*, 347–356, doi:10.1007/s10162-015-0508-x.

14. Brand, Y.; Levano, S.; Radojevic, V.; Naldi, A.M.; Setz, C.; Ryan, A.F.; Pak, K.; Hemmings, B.A.; Bodmer, D. All Akt Isoforms (Akt1, Akt2, Akt3) Are Involved in Normal Hearing, but Only Akt2 and Akt3 Are Involved in Auditory Hair Cell Survival in the Mammalian Inner Ear. *PLoS One* **2015**, *10*, e0121599, doi:10.1371/journal.pone.0121599.

15. Fu, X.; Sun, X.; Zhang, L.; Jin, Y.; Chai, R.; Yang, L.; Zhang, A.; Liu, X.; Bai, X.; Li, J.; et al. Tuberous Sclerosis Complex-Mediated MTORC1 Overactivation Promotes Age-Related Hearing Loss. *J Clin Invest* **2018**, *128*, 4938–4955, doi:10.1172/JCI98058.

16. Fetoni, A.R.; Eramo, S.L.M.; Paciello, F.; Rolesi, R.; Samengo, D.; Paludetti, G.; Troiani, D.; Pani, G. The Redox Protein P66(Shc) Mediates Cochlear Vascular Dysfunction and Transient Noise-Induced Hearing Loss. *Sci Rep* **2016**, *6*, 25450, doi:10.1038/srep25450.

17. Saxton, T.M.; Cheng, A.M.; Ong, S.H.; Lu, Y.; Sakai, R.; Cross, J.C.; Pawson, T. Gene Dosage-Dependent Functions for Phosphotyrosine-Grb2 Signaling during Mammalian Tissue Morphogenesis. *Curr Biol* **2001**, *11*, 662–670, doi:10.1016/s0960-9822(01)00198-1.

18. Powell, M.B.; Hyman, P.; Bell, O.D.; Balmain, A.; Brown, K.; Alberts, D.; Bowden, G.T. Hyperpigmentation and Melanocytic Hyperplasia in Transgenic Mice Expressing the Human T24 Ha-Ras Gene Regulated by a Mouse Tyrosinase Promoter. *Mol Carcinog* **1995**, *12*, 82–90, doi:10.1002/mc.2940120205.

19. Wojnowski, L.; Stancato, L.F.; Zimmer, A.M.; Hahn, H.; Beck, T.W.; Larner, A.C.; Rapp, U.R.; Zimmer, A. Craf-1 Protein Kinase Is Essential for Mouse Development. *Mech Dev* **1998**, *76*, 141–149, doi:10.1016/s0925-4773(98)00111-7.

20. de Iriarte Rodríguez, R.; Magariños, M.; Pfeiffer, V.; Rapp, U.R.; Varela-Nieto, I. C-Raf Deficiency Leads to Hearing Loss and Increased Noise Susceptibility. *Cell Mol Life Sci* **2015**, *72*, 3983–3998, doi:10.1007/s00018-015-1919-x.

21. Fernández-Medarde, A.; Barhoum, R.; Riquelme, R.; Porteros, A.; Núñez, A.; de Luis, A.; de Las Rivas, J.; de la Villa, P.; Varela-Nieto, I.; Santos, E. RasGRF1 Disruption Causes Retinal Photoreception Defects and Associated Transcriptomic Alterations. *J Neurochem* **2009**, *110*, 641–652, doi:10.1111/j.1471-4159.2009.06162.x.

22. Kurioka, T.; Matsunobu, T.; Satoh, Y.; Niwa, K.; Endo, S.; Fujioka, M.; Shiotani, A. ERK2 Mediates Inner Hair Cell Survival and Decreases Susceptibility to Noise-Induced Hearing Loss. *Sci Rep* **2015**, *5*, 16839, doi:10.1038/srep16839.

23. Celaya, A.M.; Sánchez-Pérez, I.; Bermúdez-Muñoz, J.M.; Rodríguez-de la Rosa, L.; Pintado-Berninches, L.; Perona, R.; Murillo-Cuesta, S.; Varela-Nieto, I. Deficit of Mitogen-Activated Protein Kinase Phosphatase 1 (DUSP1) Accelerates Progressive Hearing Loss. *Elife* **2019**, *8*, doi:10.7554/eLife.39159.

24. Bermúdez-Muñoz, J. M.; Celaya, A. M.; García_Mato, A.; Muñoz-Espín, D.; Rodríguez-de la Rosa, L.; Serrano, M.; Varela-Nieto, I. Dual-Specificity Phosphatase 1 (DUSP1) Has a Central Role in Redox Homeostasis and Inflammation in the Mouse Cochlea. *Antioxidants* **2021**, *In the press*.
