# Supplementary material for: Insulin-like Growth Factor 1 Signaling in Mammalian Hearing
Source: Genes (Basel). 2021 Sep 29;12(10):1553. doi: 10.3390/genes12101553 (PMC8535591; doi:10.3390/genes12101553)
Supplement: Supplementary file 1 [file genes-12-01553-s001.zip › genes-13605331_Table S2.pdf]

Syndrome	OMIM /ORPHA	*Gene* (Inheritance)	Phenotype	Biochemical data
**CPHD1**	613038 /95494	*POU1F1* (AD, AR)	Severe growth deficiency, distinctive facial features (prominent forehead, marked midfacial hypoplasia, depressed nasal bridge, deep-set eyes, short nose and anteverted nostrils), hypoplastic pituitary gland and severe mental retardation.	Low or absent GH, TSH and PRL.
**CPHD2**	262600 /95494	*PROP1* (AR)	Severe growth retardation, proportionate short stature, frontal bossing and blue sclera.	Deficient GH, IGF-1, TSH, PRL, LH and FSH.
**CPHD3**	221750 /231720	*LHX3* (AR)	Short stature, abnormal pituitary morphology, mental retardation, restricted neck rotation and **sensorineural hearing loss** [1].	Low or absent GH, TSH, FSH, LH, PRL and ACTH.
**CPHD4**	262700/95 494	*LHX4* (AD)	Short stature, delayed bone age, hypothyroidism, hypoglycaemia.	Low or absent GH, TSH, FSH, LH and ACTH.
**IGHD1A**	262400 /231662	*GH1* (AR)	Severe growth failure, baby doll facies and good response to rhGH treatment but tendency to develop antibodies [2]..	Isolated GH deficiency and hypoglycaemia.
**IGHD1B**	612781 /231671	*GH1* (AR)	Short stature, delayed bone age and good response to rhGH treatment but without antibody formation.	Low level of GH.
**IGHD2**	173100 /231679	*GH1* (AD)	Variable height deficit and age at presentation, and good response to rhGH. Patients may show anterior pituitary hypoplasia on MRI.	Low level of GH, insulin response to glucose greater than normal.
**IGHD3**	307200 /231692	*BTK* (X-linked recessive)	Short stature, delayed bone age, good response to treatment with rhGH. Agammaglobulinemia, reduced numbers of B cells and frequent infections (sinusitis, conjunctivitis, otitis media and **hearing loss**, pneumonia, hepatitis, urinary tract infections, arthritis and meningitis).	Deficient GH response to insulin, arginine, or levodopa.
**IGHD4**	618157 /231671	*GHRHR* (AR)	Early and severe growth failure, proportionate short stature, truncal obesity, frontal bossing, delayed bone age and high-pitched voice. Blunted GH response to different provocation tests and good response to GH therapy. Patients can present mild high-tones **sensorineural hearing loss** [3], reduced transient evoked optoacoustic emissions and stapedial reflex.	Low level of IGF-1 and IGFBP-3.
**IGHD5**	618160 /231662	*RNPC3* (AR)	Severe postnatal growth failure, short stature, truncal obesity, microcephaly, frontal bossing, delayed bone maturation, and hypoplasia of the anterior pituitary. Patients respond well to GH therapy.	Low level of GH and elevated ghrelin level.
**Kowarski syndrome**	262650 /629	*GH1* (AR)	Growth retardation, dwarfism and delayed bone age.	Low IGF-1 level.
**IGHD due to defect in GHRH**	ND	*GHRH* (ND)	Presumably *GHRH* is mutant in some cases of IGHD. The phenotype is similar to IGHD, but patients respond to the administration of GHRH.	Low level of GH.
**Partial IGHD**	615925 /314811	*GHSR* (AD, AR)	Short stature, postnatal growth delay and delayed bone age in some patients.	Partial GH deficiency, low level of IGF-1 and low GH response.

**Partial GHI**	604271 /314802	*GHR* (AD)	Short stature and delayed bone age in some patients. Response to GH therapy.	Low level of GH-binding protein and extremely low IGF-1 level.
**Laron syndrome**	262500 /633	*GHR* (AR)	Marked short stature, childlike body proportions in adults, small facies, delayed bone maturation, short limbs, hip degeneration and high-pitched voice. Untreated patients have **sensorineural hearing loss** [4] (low-tone, high-tone, or combined high-/low-tone) and auditory hypersensitivity.	Normal or increased GH level and low IGF-1 level.
**GHISID1**	245590 /220465	*STAT5B* (AR)	Short stature, failure to thrive, prominent forehead, saddle nose, high-pitched voice, delayed bone age, delayed puberty, immune dysregulation, including chronic pulmonary disease, interstitial pneumonitis, recurrent or severe infections, eczema and autoimmune arthritis.	Decreased IGF-1, IGFBP3, and ALS.
**GHISID2**	618985 /220465	*STAT5B* (AD)	Short stature, delayed bone age, microcephaly, delayed puberty, mild immune dysregulation, recurrent infections, increased serum IgE, asthma, or celiac disease.	Decreased IGF-1.
**Primary IGF-1 deficiency**	608747 /73272	*IGF1* (AR)	All patients show growth retardation. Other symptoms like cognitive delay and **sensorineural hearing loss** depends on the mutation (See Table 1).	Decreased IGF-1 level and increased GH level.
**Primary IGF-2 deficiency (Silver-Russell syndrome-3**	616489 /813	*IGF2* (AD)	Severe prenatal and postnatal growth restriction, short stature, low weight, delayed bone age, delayed puberty, relative macrocephaly, dysmorphic facial features (triangular face, prominent forehead, micrognatia, low-set ears and preauricular fistulas), **sensorineural deafness** [5], limb defects, genitourinary (micropenis, hypospadia, cryptochidism) and cardiovascular (persistent ductus arteriosus, atrial and ventricular septal defects) anomalies.	Normal or slightly elevated GH level, normal to high serum IGF-1 levels, low IGF2 level, and normal to high serum IGFBP3 level.
**Primary ALS deficiency**	615961 /140941	*IGFALS* (AR)	Mild growth retardation, short stature, delayed bone age, decreased bone mineral density, pubertal delay, insulin insensitivity, and poor response to rhGH.	Severely reduced IGF-1 and IGFBP3 levels and increased insulin.
**Resistance to IGF-1**	270450 /73273	*IGF1R* (AD, AR)	Intrauterine growth retardation, postnatal growth failure, delayed bone age, short stature, low weight, microcephaly, facial dysmorphism (triangular face, micrognathia, low-set ears, deep-set eyes, broad nasal bridge, small mouth, delayed eruption of dentition and pterygium colli), cardiovascular anomalies and **sensorineural hearing loss** [6] .	Increased IGF-1, increased or normal GH, and elevated baseline insulin.
**Donohue syndrome**	246200 /508	*INSR* (AR)	Prenatal and postnatal growth retardation, severe failure to thrive, dysmorphic facial features (small face, large ears, wide nostrils and large mouth), delayed bone age, breats hiperplasia and large genital organs. Death often in early infancy.	Hyperinsulinemia, postprandial hyperglycaemia and fasting hypoglycaemia.
**Rabson-Mendenhall syndrome**	262190 /769	*INSR* (AR)	Short stature, low weight, small for gestational age, coarse facies, prognathism, large and fissured tongue, dental displasia and large genotal organs. Survival to 5-15 years of age.	Hyperinsulinemia, insulin resistant DM, diabetic ketoacidosis, and altered melatonin secretion.
**SHORT syndrome**	269880 /3163	*PIK3R1* (AD)	**S**hort stature, hyperextensibility of joints or inguinal hernia, ocular depression, rieger anomaly, and teething delay. Low birth length and weight, recognizable facial gestalt (triangular facies, chin dimple, micrognathia, prominent forehead, deep-set eyes, lack of facial fat, hypoplastic nasal alae, wide nasal bridge) and **sensorineural deafness** [7–11].	Insulin resistant DM and hyperglycemia

**Noonan syndrome-1**	163950 /648	*PTPN11* (AD)	Short stature, facial dysmorphism (broad forehead, hypertelorism, downslanting palpebral fissures, high-arched palate, low-set and posteriorly rotated ears), congenital heart defects (pulmonic stenosis and hypertrophic cardiomyopathy are the most common), skeletal defects (chest and spine deformities), webbed neck, mental retardation, cryptorchidism, bleeding diathesis. Most of the patients present **sensorineural and/or conductive deafness,** mainly bilateral severe **deafness** [12–14].	Partial deficiency of factor XI, XII, XIII and thrombocytopenia.
**Noonan syndrome-3**<br>**Noonan syndrome-4**<br>**Noonan syndrome-5**	609942 /648<br>610733 /648<br>611553 /648	*KRAS* (AD)<br>*SOS1* (AD)<br>*RAF1* (AD)	Short stature, macrocephaly, dolichocephaly (in some patients), Prominent forehead and chin, low-set ears and thickened helix, downslanting palpebral fissures and hypertelorism, Short neck, hypertrophic cardiomyopathy, cryptorchidism, mental retardation. Some patients show **sensorineural and/or conductive deafness** [14].	Partial deficiency of factor XI and XIII
**LEOPARD syndrome-1**	151100 /500	*PTPN11* (AD)	Lentigines, EKG abnormalities, ocular hypertelorism, obstructive cardiomyopathy, pulmonic stenosis, abnormalities of genitalia, retardation of growth, facial features (triangular face, prognathism, low-set ears, broad and flat nose, cleft palate, pterygium colli) and severe bilateral **sensorineural deafness** [15–17].	Low levels of FSH, LH and TSH in some patients with endocrine abnormalities.
**LEOPARD syndrome-2**	611554 /500	*RAF1* (AD)	Short stature, hypertrophic cardiomyopathy, lentigines and *cafe au lait* spots, craniofacial anomalies (dolichocephaly, downslanting palpebral fissures, hypertelorism, thick lips, low-set ears and prominent chin), short webbed neck, cubitus valgus, and delayed puberty.	
**LEOPARD syndrome-3**	613707 /500	*BRAF* (AD)	Poor growth, short stature, craniofacial anomalies (low set ears, depressed nasal bridge), short and webbed neck, mitral and aortic valve dysplasia, cognitive deficits, neonatal hypotonia and seizures, and **sensorineural deafness** [18].	
**IMAGE syndrome**	614732 /85173	*CDKN1C* (AD)	Intrauterine growth retardation, metaphyseal dysplasia, adrenal hypoplasia and genital anomalies. Growth failure, delayed bone age, macrocephaly, prominent forehead, low-set ears, short nose and flat nasal bridge**. Sensorineural hearing loss** has been described in one case [19].	GH deficiency and hypercalcemia
**Dauber-Argente type of short stature**	619489	*PAPPA2* (AR)	Postnatal growth retardation, mild to severe short stature, microcephaly, triangular face, small chin, and delayed bone age.	Elevated total IGF1 but reduced free IGF1. Elevated IGF2, IGFBP3, IGFBP5 and IGFALS
**Turner syndrome**	/881	Total/partial absence of Chr. X	Short stature, ovarian failure, variable onset depending on the chromosomal anomalies. Lymphoedema, bone, cardiovascular, thyroid and gastrointestinal anomalies. More than 50% of the patients show **sensorineural hearing loss** (more frequent in karyotypes with a loss of the short p-arm on the X-chromosome), although the mechanism is not known (estrogen deficiency, cell cycle delay or IGF-1 deficiency [20]). GH–IGF–IGFBP axis disturbed, with a partly normalizing effect of sex hormone replacement therapy [21].	Normal level of IGF-1 and IGFBPs 1, 2 and 3. IGFBP-3 ternary complex was significantly reduced

Table S2. Clinical conditions with GH/IGF-1 axis alterations. Non-exhaustive list of reported rare syndromes showing alterations in the GH/IGF-1 axis and hearing loss (in bold) or without reported data on the auditory function of patients. Abbreviations: ACTH, adrenocorticotropic hormone; AD, autosomal dominant; ADMIO, infantile-onset multisystem autoimmune disease-1; ALS, acid-labile subunit; AR, autosomal recessive; CPHD, combined pituitary hormone deficiency; Chr., chromosome; DM, diabetes mellitus; IGHD, isolated growth hormone deficiency; FSH, follicle stimulation hormone; GH, growth hormone; GHI, growth hormone insensitivity; GHISID, growth hormone insensitivity syndrome with immune dysregulation; IgE, immunoglobulin E; LH, luteinizing hormone; MRI, magnetic resonance imaging; ND, not determined; PRL, prolactin; rhGH, recombinant human growth hormone therapy; TSH, Thyroid-stimulating hormone. Source of information: Online Mendelian Inheritance in Man (OMIM) Catalogue available at https://www.omim.org/; Orphanet Database available at https://www.orpha.net/consor/cgi-bin/index.php?lng=EN.

References

1. Ramzan, K.; Bin-Abbas, B.; Al-Jomaa, L.; Allam, R.; Al-Owain, M.; Imtiaz, F. Two Novel LHX3 Mutations in Patients with Combined Pituitary Hormone Deficiency Including Cervical Rigidity and Sensorineural Hearing Loss. *BMC Endocr Disord* **2017**, *17*, 17, doi:10.1186/s12902-017-0164-8.
2. Gómez, J.G.; Devesa, J. Growth Hormone and the Auditory Pathway: Neuromodulation and Neuroregeneration. *Int J Mol Sci* **2021**, *22*, 2829, doi:10.3390/ijms22062829.
3. Prado-Barreto, V.M.; Salvatori, R.; Santos Júnior, R.C.; Brandão-Martins, M.B.; Correa, E.A.; Garcez, F.B.; Valença, E.H.O.; Souza, A.H.O.; Pereira, R.M.C.; Nunes, M.A.P.; et al. Hearing Status in Adult Individuals with Lifetime, Untreated Isolated Growth Hormone Deficiency. *Otolaryngol Head Neck Surg* **2014**, *150*, 464–471, doi:10.1177/0194599813517987.
4. Attias, J.; Zarchi, O.; Nageris, B.I.; Laron, Z. Cochlear Hearing Loss in Patients with Laron Syndrome. *Eur Arch Otorhinolaryngol* **2012**, *269*, 461–466, doi:10.1007/s00405-011-1668-x.
5. Bigoni, S.; Mauro, A.; Ferlini, A.; Corazzi, V.; Ciorba, A.; Aimoni, C. Cochlear Malformation and Sensorineural Hearing Loss in the Silver-Russell Syndrome. *Minerva Pediatr* **2018**, *70*, 638–639, doi:10.23736/S0026-4946.17.04993-3.
6. Ester, W.A.; van Duyvenvoorde, H.A.; de Wit, C.C.; Broekman, A.J.; Ruivenkamp, C.A.L.; Govaerts, L.C.P.; Wit, J.M.; Hokken-Koelega, A.C.S.; Losekoot, M. Two Short Children Born Small for Gestational Age with Insulin-like Growth Factor 1 Receptor Haploinsufficiency Illustrate the Heterogeneity of Its Phenotype. *J Clin Endocrinol Metab* **2009**, *94*, 4717–4727, doi:10.1210/jc.2008-1502.
7. Toriello, H.V.; Wakefield, S.; Komar, K.; Higgins, J.V.; Waterman, D.F. Report of a Case and Further Delineation of the SHORT Syndrome. *Am J Med Genet* **1985**, *22*, 311–314, doi:10.1002/ajmg.1320220214.
8. Schwingshandl, J.; Mache, C.J.; Rath, K.; Borkenstein, M.H. SHORT Syndrome and Insulin Resistance. *Am J Med Genet* **1993**, *47*, 907–909, doi:10.1002/ajmg.1320470619.
9. Bankier, A.; Keith, C.G.; Temple, I.K. Absent Iris Stroma, Narrow Body Build and Small Facial Bones: A New Association or Variant of SHORT Syndrome? *Clin Dysmorphol* **1995**, *4*, 304–312, doi:10.1097/00019605-199510000-00005.
10. Brodsky, M.C.; Whiteside-Michel, J.; Merin, L.M. Rieger Anomaly and Congenital Glaucoma in the SHORT Syndrome. *Arch Ophthalmol* **1996**, *114*, 1146–1147, doi:10.1001/archopht.1996.01100140348022.
11. Avila, M.; Dyment, D.A.; Sagen, J.V.; St-Onge, J.; Moog, U.; Chung, B.H.Y.; Mo, S.; Mansour, S.; Albanese, A.; Garcia, S.; et al. Clinical Reappraisal of SHORT Syndrome with PIK3R1 Mutations: Toward Recommendation for Molecular Testing and Management. *Clin Genet* **2016**, *89*, 501–506, doi:10.1111/cge.12688.
12. Ziegler, A.; Loundon, N.; Jonard, L.; Cavé, H.; Baujat, G.; Gherbi, S.; Couloigner, V.; Marlin, S. Noonan Syndrome: An Underestimated Cause of Severe to Profound Sensorineural Hearing Impairment. Which Clues to Suspect the Diagnosis? *Otol Neurotol* **2017**, *38*, 1081–1084, doi:10.1097/MAO.0000000000001509.
13. van Nierop, J.W.I.; van Trier, D.C.; van der Burgt, I.; Draaisma, J.M.T.; Mylanus, E.A.M.; Snik, A.F.; Admiraal, R.J.C.; Kunst, H.P.M. Cochlear Implantation and Clinical Features in Patients with Noonan Syndrome and Noonan Syndrome with Multiple Lentigines Caused by a Mutation in PTPN11. *Int J Pediatr Otorhinolaryngol* **2017**, *97*, 228–234, doi:10.1016/j.ijporl.2017.04.024.
14. van Trier, D.C.; van Nierop, J.; Draaisma, J.M.T.; van der Burgt, I.; Kunst, H.; Croonen, E.A.; Admiraal, R.J.C. External Ear Anomalies and Hearing Impairment in Noonan Syndrome. *Int J Pediatr Otorhinolaryngol* **2015**, *79*, 874–878, doi:10.1016/j.ijporl.2015.03.021.

15. Martínez-Quintana, E.; Rodríguez-González, F. LEOPARD Syndrome Caused by Tyr279Cys Mutation in the PTPN11 Gene. *Mol Syndromol* **2012**, *2*, 251–253, doi:10.1159/000335995.
16. Kim, J.; Kim, M.R.; Kim, H.J.; Lee, K.-A.; Lee, M.-G. LEOPARD Syndrome with PTPN11 Gene Mutation Showing Six Cardinal Symptoms of LEOPARD. *Ann Dermatol* **2011**, *23*, 232–235, doi:10.5021/ad.2011.23.2.232.
17. Chu, H.-S.; Chung, H.-S.; Ko, M.-H.; Kim, H.-J.; Ki, C.-S.; Chung, W.-H.; Cho, Y.-S.; Hong, S.H. Syndromic Hearing Loss in Association with PTPN11-Related Disorder: The Experience of Cochlear Implantation in a Child with LEOPARD Syndrome. *Clin Exp Otorhinolaryngol* **2013**, *6*, 99–102, doi:10.3342/ceo.2013.6.2.99.
18. Sarkozy, A.; Carta, C.; Moretti, S.; Zampino, G.; Digilio, M.C.; Pantaleoni, F.; Scioletti, A.P.; Esposito, G.; Cordeddu, V.; Lepri, F.; et al. Germline BRAF Mutations in Noonan, LEOPARD, and Cardiofaciocutaneous Syndromes: Molecular Diversity and Associated Phenotypic Spectrum. *Hum Mutat* **2009**, *30*, 695–702, doi:10.1002/humu.20955.
19. Balasubramanian, M.; Sprigg, A.; Johnson, D.S. IMAGe Syndrome: Case Report with a Previously Unreported Feature and Review of Published Literature. *Am J Med Genet A* **2010**, *152A*, 3138–3142, doi:10.1002/ajmg.a.33716.
20. Bonnard, Å.; Bark, R.; Hederstierna, C. Clinical Update on Sensorineural Hearing Loss in Turner Syndrome and the X-Chromosome. *Am J Med Genet C Semin Med Genet* **2019**, *181*, 18–24, doi:10.1002/ajmg.c.31673.
21. Gravholt, C.H.; Chen, J.-W.; Oxvig, C.; Overgaard, M.T.; Christiansen, J.S.; Frystyk, J.; Flyvbjerg, A. The GH-IGF-IGFBP Axis Is Changed in Turner Syndrome: Partial Normalization by HRT. *Growth Horm IGF Res* **2006**, *16*, 332–339, doi:10.1016/j.ghir.2006.09.001.
